# Listeria-vectored multi-antigenic tuberculosis vaccine protects C57BL/6 and BALB/c mice and guinea pigs against *Mycobacterium tuberculosis* challenge

**DOI:** 10.1038/s42003-022-04345-1

**Published:** 2022-12-20

**Authors:** Qingmei Jia, Saša Masleša-Galić, Susana Nava, Marcus A. Horwitz

**Affiliations:** Division of Infectious Diseases, Department of Medicine, 552-215 Center for Health Sciences, School of Medicine, University of California – Los Angeles, 10833 Le Conte Avenue, Los Angeles, CA 90095-1688 USA

**Keywords:** Preclinical research, Live attenuated vaccines, Live attenuated vaccines, Tuberculosis

## Abstract

*Mycobacterium tuberculosis* (Mtb) infects one-third of the world’s population and is a leading cause of death from a single infectious agent. New TB vaccines are urgently needed to augment immunity conferred by the current modestly protective BCG vaccine. We have developed live attenuated recombinant *Listeria monocytogenes* (rLm)-vectored TB vaccines expressing five [Mpt64/23.5-EsxH/TB10.4-EsxA/ESAT6-EsxB/CFP10-Ag85B/r30] (rLmMtb5Ag) or nine (additionally EsxN-PPE68-EspA-TB8.4) immunoprotective Mtb antigens (rLmMtb9Ag) and evaluated them for safety, immunogenicity and efficacy as standalone vaccines in two mouse models and an outbred guinea pig model. In immunogenicity studies, rLmMtb5Ag administered subcutaneously induces significantly enhanced antigen-specific CD4+ and CD8+ T-cell responses in C57BL/6 and BALB/c mice, and rLmMtb9Ag induces antigen-specific CD4+ and CD8+ T-cell proliferation in guinea pigs. In efficacy studies, both rLmMtb5Ag and rLmMtb9Ag are safe and protect C57BL/6 and BALB/c mice and guinea pigs against aerosol challenge with highly virulent Mtb. Hence, multi-antigenic rLm vaccines hold promise as new vaccines against TB.

## Abstract (web summary)

An attenuated Listeria monocytogenes vectored multiantigenic vaccine is developed, which offers protection against Mycobacterium tuberculosis in mice and guinea pigs.

## Introduction

*Mycobacterium tuberculosis* (Mtb), the leading cause of death from a single infectious agent, at least until the COVID-19 pandemic, infects approximately one-third of the world’s population and causes active tuberculosis (TB) in approximately 10.4 million people and death in ~1.7 million people annually^1^. The only currently approved TB vaccine, bacille Calmette-Guérin or BCG, has been administered to ~5 billion people on earth; it provides only moderate protection against childhood TB and highly variable protection (0–87%) against adult pulmonary TB. Hence new TB vaccines are a very high global public health priority and could play an important role in achieving the World Health Organization (WHO) End TB Strategy target of a 95% reduction in TB deaths and a 90% reduction in TB incidence by 2035 compared to 2015^2^.

While most people in the world are vaccinated with BCG in infancy, especially in low- and middle-income countries where the incidence of TB is highest, most cases of TB occur in adults. In 2020, 89% of new cases of TB occurred in people over 14 years of age^2^. Thus, an effective TB vaccine is needed to boost the immunity of adolescents and adults. In support of this strategy, an impact and cost-effectiveness study of potential TB vaccines in low- and middle-income countries indicated that a vaccine targeting adolescents and adults could have a greater impact than one targeting infants^3^.

Two types of new TB vaccines are under development – prime vaccines to replace BCG and booster vaccines to enhance the immunity of people previously vaccinated with BCG or in the future vaccinated with replacement vaccines for BCG. Prime vaccines under development, all comprising mycobacteria, include rBCG30, the first vaccine demonstrated more potent than BCG and the first potential replacement vaccine for BCG to enter human trials^4–7^; rBCG(*mbtB*)30, the first vaccine demonstrated more potent and safer than BCG^8^; MTBVAC, an attenuated Mtb^9^, and VPM-1002, a modified BCG^10^. TB booster vaccines under development, all subunit vaccines, include protein/adjuvant vaccines such as r30 (Antigen 85B), the first defined booster vaccine against TB^4^, H1 (Antigen 85B – ESAT6)^11,12^, H4 (Antigen 85B-TB10.4)^13,14^, M72^15,16^, and ID93^17,18^; virus vectored vaccines such as MVA85A^19,20^; and bacterium vectored vaccines including Salmonella-vectored vaccines^21^ and Listeria-vectored vaccines, including rLm30^22^ and rLmMtb5Ag^23^, as well as rLmMtb9Ag, described herein.

Targeting an adolescent and adult population for a new TB booster vaccine has practical implications regarding the preclinical development and testing of such a vaccine. Since most people in the world in need of an improved TB vaccine have been vaccinated with BCG in infancy, a new TB booster vaccine should demonstrate efficacy in enhancing the protective immunity conferred by BCG in preclinical animal models of pulmonary TB. But if the intended targets of the new TB booster vaccine are adolescents and adults, in whom immunity from BCG vaccination in infancy has largely waned, then a new TB booster vaccine ideally would additionally demonstrate protective efficacy as a standalone vaccine in pre-clinical animal models of pulmonary TB.

Our candidate TB booster vaccine comprises a live attenuated replicating *Listeria monocytogenes* (Lm) bacterium expressing immunoprotective Mtb antigens. The Lm vector was chosen in large part because of its ability to induce robust antigen-specific CD4+ and CD8+ T cell responses to expressed recombinant antigens − both types of T cell immunity are central to immunoprotection against Mtb. The wild-type parent of this vector, a fast growing, Gram-positive, facultative intracellular bacterium that occasionally infects humans and can cause food-borne disease outbreaks, shares important features of its intracellular lifestyle with Mtb. Like Mtb, upon entry into host cells, which include mononuclear phagocytes, Lm initially resides in a phagosome, a site favoring antigen presentation via class II MHC molecules and the induction of antigen-specific CD4+ T cells. Subsequently, also in common with Mtb, Lm escapes the phagosome and multiplies in the host cytosol, a site favoring antigen presentation via class I MHC molecules and the induction of antigen-specific CD8+ T cells. As a result of its capacity to induce long-lived cell-mediated immune responses, genetically attenuated Lm has been developed as a vaccine vector for cancer and infectious diseases^24^.

The specific Lm vector that we employ, Lm Δ*actA* ΔinlB prfA* (Lm Δ*actA* Δ*inlB* ΔuvrAB prfA*)^22,25^ has been attenuated from wild-type Lm and rendered more effective as a vaccine vector via several genetic manipulations^25^. First, *actA*, a gene encoding the cell surface transmembrane protein ActA, which promotes intracellular motility via actin polymerization, has been deleted. ActA deletional mutants are able to grow within the cytosol of infected cells, but are unable to induce cell-to-cell spread, resulting in ~1000-fold attenuation in virulence in mouse models^24^. Second, a deletion of *inlB*, encoding internalin B, a virulence factor that promotes invasion of various mammalian cells including epithelial cells, endothelial cells and hepatocytes, inhibits Lm uptake into non-phagocytic cells, such as hepatocytes, but not into phagocytic cells, including antigen-presenting cells; hence, in the double deletional Δ*actA* Δ*inlB* mutant, off-target toxicity is minimized but not antigen presentation of secreted recombinant antigens in antigen presenting cells^26^. Third, a point mutation (G155S) in the master virulence regulator PrfA that renders it constitutively active, promotes Lm escape into the host cell cytosol, and as a result of upregulated expression of PrfA and PrfA-dependent genes, shows enhanced expression of downstream recombinant proteins^25,27,28^.

Using Lm Δ*actA* ΔinlB prfA* as a vaccine vector, we have developed several recombinant Lm-vectored Mtb vaccines (rLm) including rLm30^22^, rLmMtb5Ag (rLm5Ag)^23^, and in this study, rLmMtb9Ag (rLm9Ag). The rLm30 vaccine expresses a single Mtb antigen – the 30-kDa major secretory protein or Antigen 85B (r30 or Ag85B, gene Rv1886); rLm5Ag expresses a fusion protein of 5 Mtb antigens − Mpt64/23.5 (Rv1980c), EsxH/TB10.4 (Rv0288), EsxA/ESAT6 (Rv3875), EsxB/CFP10 (Rv3874) and r30; and rLm9Ag expresses, in addition to the 5 antigens expressed by rLm5Ag, a fusion protein comprising 4 additional Mtb antigens - EspA (Rv3616c), EsxN (Rv1793), PPE68 (Rv3873) and TB8.4 (Rv1174c). These 4 additional proteins were selected on the basis of their capacity, when administered as part of a rLm booster vaccine, to enhance protective immunity against Mtb aerosol challenge in BCG-immunized mice^23^. In previous studies of rLm30 and rLm5Ag, we have shown that i) immunization of mice with BCG has no significant effect on local replication or systemic dissemination, growth, and clearance of rLm30 administered intradermally 12 or 15 weeks later^22,23^; ii) boosting BCG-primed mice with rLm30 and rLm5Ag enhances Mtb antigen-specific CD4+ and CD8+ T cell-mediated immune responses^22,23^; and iii) boosting BCG-primed C57BL/6 and BALB/c mice with rLm30 and rLm5Ag enhances protective immunity against aerosolized Mtb^22,23^.

Herein, we investigate the immunogenicity and efficacy of rLm5Ag and rLm9Ag as standalone vaccines in three animal models of pulmonary TB–inbred C57BL/6 and BALB/c mice and outbred Hartley guinea pigs. We test them as standalone vaccines - not because we envision them as replacement vaccines for BCG but because, as noted above, most of the people in the world in need of a TB booster vaccine were vaccinated with BCG in infancy; hence, their BCG-induced immunity will have largely waned by the time they would receive a TB booster vaccine many years and often decades later. As testing the potency of a heterologous booster vaccine administered decades after a prime vaccine is not feasible in small animal models, we elected instead to test the vaccines as standalone vaccines so as to mimic the situation in which BCG-induced immunity has completely waned. In so doing, we examined two different strains of mice, C57BL/6 and BALB/c, because these mice display different innate and acquired immune responses to infection, including mycobacterial infection with BCG^29,30^. We additionally examined guinea pigs because these animals develop disease more akin to that of humans than do most strains of mice; e.g., they are highly susceptible to clinical disease after low dose infection with Mtb; they show strong cutaneous delayed-type hypersensitivity to tuberculin; and they display Langhans giant cells in lung lesions and develop caseating granulomas^31^.

In C57BL/6 and BALB/c mice, we show that homologous priming-boosting with rLm5Ag and rLm9Ag vaccines induces antigen-specific CD4+ and CD8+ T cell immune responses and protective immunity against aerosol challenge with virulent Mtb. In guinea pigs, we show that homologous priming-boosting with rLm9Ag induces Mtb antigen-specific lymphocyte proliferation and elevated frequencies of CD8+ T cells in the lungs and/or spleens, and that immunization with rLm5Ag or rLm9Ag induces significant protective immunity against Mtb aerosol challenge.

## Results

### Construction and verification of rLm9Ag expressing fusion proteins of Mtb5Ag and Mtb5AgII from the *comK* and *tRNA*^arg^ loci, respectively

**Fig. 1:**
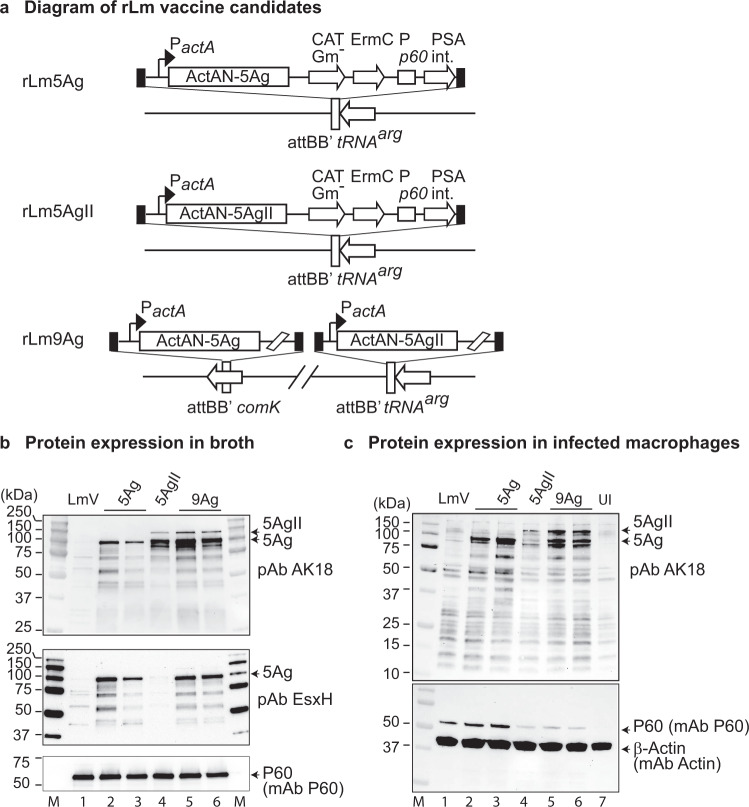
Construction and verification of rLm9Ag vaccine. **a** Diagram of rLm5Ag and rLm9Ag vaccine candidates. Expression cassettes ActAN-5Ag for rLm5Ag (top panel) and ActAN-5AgII for rLm5AgII (middle panel) driven by the Lm *actA* promoter were integrated at the *tRNA*^*arg*^ loci of the corresponding rLm chromosome*;* expression cassettes ActAN-5Ag and ActAN-5AgII for rLm9Ag were integrated at the *comK* and *tRNA*^*arg*^ loci of the Lm chromosome, respectively (bottom panel). **b** Expression of heterologous and homologous proteins by rLm vaccine candidates grown in broth medium. Stocks of rLm vaccine candidates were inoculated into Brain Heart Infusion (BHI) medium and grown overnight at 37 °C with agitation. The overnight culture was collected by centrifugation, resuspended in PBS supplemented with Halt Proteinase Inhibitor (Thermo Fisher Scientific), and lysed in SDS buffer. Equivalent amounts of bacterial lysates of each rLm were analyzed by Western blotting sequentially using rabbit polyclonal antibody to ActAN (pAb AK18) (top panel), rabbit polyclonal antibody to Mtb EsxH (middle panel); and monoclonal antibody to Lm P60 (serving as a loading control). The membrane was stripped before re-probing. **c** The murine macrophage-like (J774A.1) cells were uninfected (UI) or infected at a MOI of 10 with LmVector or an rLm vaccine expressing Mtb 5Ag, 5AgII, or 9Ag that had been grown to stationary phase. At 5.5 h post-infection, the infected cells were lysed and subjected to Western blotting using polyclonal antibody AK18 (upper panel); the membrane was stripped and re-probed with a monoclonal antibody to Lm P60 and a monoclonal antibody to cellular protein β-actin (lower panel), as indicated on the right border of each panel. In both panels **b** and **c**: M, protein standards; Lane 1, LmVector (LmV); Lanes 2 and 3, two clones of rLm5Ag expressing ActAN-Mpt64-EsxH-EsxA-EsxB-r30 from the *tRNA*^*arg*^ locus; Lane 4, rLm5AgII expressing ActAN-Mpt64-EsxN-PPE68-EspA-TB8.4 from the *tRNA*^*arg*^ locus; Lanes 5 and 6, two clones of rLm9Ag expressing a total of 9 different antigens, ActAN-5Ag from the *comK* locus and ActAN-5AgII from the *tRNA*^*arg*^ locus; lane 7 (Panel c only), uninfected cells. On the left border of each panel are listed the sizes (kDa) of the molecular mass standards. Proteins of interests are indicated with arrows to the right of the protein band. Estimated Mw of ActAN-5AgII: 118-kDa; ActAN-5Ag: 94-kDa; Lm P60: 60 kDa; and β-actin, 42 kDa. The full-length images for b and c are shown in Supplementary Fig. 1.

To construct rLm5Ag (expressing Mtb Mpt64-EsxH-EsxA-EsxB-r30, with an N-terminal fusion to ActAN, the N-terminal 100 amino acids of Lm ActA)^23^ (Supplementary Table 1) and rLm5AgII (expressing ActAN-Mpt64-EsxN-PPE68-EspA-TB8.4) (Supplementary Table 1), we integrated the pPL2e-ActAN-Mtb5Ag and pPL2e-ActAN-Mtb5AgII into the *tRNA*^*arg*^ locus of the rLm chromosome (Fig. 1a, top and middle vaccines). To construct the rLm9Ag vaccine candidate (Supplementary Table 1), we integrated pPL1-ActAN-Mtb5Ag at the *comK* locus followed by integrating pPL2e-ActAN5AgII into the *tRNA*^*arg*^ locus of the Lm chromosome (Fig. 1a, bottom vaccine). This strategy had two advantages. First, the use of two expression cassettes allowed expression of smaller fusion proteins than a single cassette expressing all 9 antigens (estimated Mw of 9-antigen fusion protein of 212 kDa vs. the estimated molecular weights of the 5Ag and 5AgII fusion proteins of 94 and 118 kDa, respectively). The smaller Mw favors better stability and expression. Second, the strategy allows use of the same leader protein of Mpt64 for both the 5AgI and 5AgII expression cassette. We have tested various combinations of Mtb fusion proteins and found that Mtb proteins fused to the C-terminus of Mpt64 tend to be expressed more abundantly than otherwise; hence Mpt64 is evidently an ideal leader protein for the expression cassette of Mtb fusion proteins. We have shown that rLm5Ag, rLm5AgII, and two clones (clones 1 and 3) of rLm9Ag express the expected fusion proteins when grown in broth medium and in infected murine macrophage-like cells. As shown in Fig. 1b and Supplementary Fig. 1 left panels, rLm9Ag vaccine clones 1 and 3 (top panel, lanes 5, 6) express both the 94-kDa 5Ag and the 118-kDa 5AgII fusion proteins (indicated by arrows), similar to the proteins expressed by rLm5AgII (lane 4) and rLm5Ag (lanes 2, 3), respectively, that are detected by the polyclonal antibody to an N-terminal peptide comprising 18 amino acids (A30-K47) of ActA (AK18, courtesy of Justin Skoble and Peter Lauer). As expected, the 94-kDa, but not the 118-kDa, protein band was also detected by the polyclonal antibody to EsxH (middle panel, lanes 2, 3, 5, and 6) which is present in the 5Ag but not the 5AgII fusion protein.

To analyze expression of the heterologous Mtb fusion proteins by rLm growing inside of macrophages, we infected murine macrophage-like cells (J774.A1) with LmVector or rLm expressing various Mtb fusion proteins at a Multiplicity of Infection (MOI) of 10. At 5.5 h post infection, we harvested the infected cells and analyzed the lysates for protein expression by SDS-PAGE and Western blotting using the polyclonal antibody AK18, which detected the predicted 94-kDa (5Ag) protein band (and non-specific protein bands) from J774A.1 cells infected with rLm5Ag, rLm5AgII, or rLm9Ag clones #1 and 3 (Fig. 1c upper panel and Supplementary Fig. 1 right upper panel, lanes 2, 3, 5, and 6, respectively) and the predicted 112-kDa (5AgII) protein band from J774A.1 cells infected with rLm5Ag I and rLm9Ag clones #1 and 3 (Fig. 1c, upper panel, lanes 4, 5, and 6, respectively), but not from LmVector-infected (upper panel, lane 1) and mock infected (upper panel, lane 7) J774A.1 cells. Antibody to Lm P60 detected a ~ 60-kDa protein band from Lm-infected (Fig. 1c lower panel and Supplementary Fig. 1 right lower panel, lanes 1–6), but not from uninfected cells (Fig. 1c, lower panel, lane 7). Antibody to β-actin detected a ~42 kDa band from uninfected and infected J774 cell lysates, as expected (Fig. 1c lower panel, and Supplementary Fig. 1 right lower panel, lanes 1–7).

### Genetic stability and growth kinetics of rLm5Ag and rLm9Ag vaccines

To evaluate the antigen expression cassette stability of rLm grown in broth culture and in infected macrophage-like cells, we examined the growth of rLm vaccine candidates in Brain Heart Infusion (BHI) broth supplemented with various antibiotics and in infected monolayers of the J774A.1 cells with stationary grown rLm vaccines and assayed bacterial replication. As shown in Supplementary Fig. 2, the rLm9Ag, rLm5Ag, and rLm5Ag I vaccine candidates grew similarly in the presence or absence of antibiotic selection, either after direct inoculation into BHI broth medium (Supplementary Fig. 2a) or after passage in murine macrophage-like cells followed by plating onto BHI agar plates supplemented with various antibiotics (Supplementary Fig. 2b, c). We verified the heterologous and homologous protein expression by Western blotting as shown in Fig. 1b, c. In addition, we passaged LmVector, rLm5Ag and rLm9Ag vaccines daily for 10 consecutive days on BHI agar plates and verified the stability of the antigen expression cassettes. We also verified the stability of the Mtb antigen expression cassettes after the rLm9Ag vaccine was passaged in guinea pigs (Supplementary Fig. 3). Thus, the antigen expression cassettes for Mtb 5Ag (Mpt64-EsxH-EsxA-EsxB-r30), Mtb 5AgII (Mpt64-EsxN-PPE68-EspA-TB8.4), and Mtb 9Ag (5Ag+ 5AgII) are stably maintained in the rLm vaccine candidates.

To examine the growth kinetics of rLm vaccine candidates in murine macrophages, we infected monolayers of J774A.1 cells with LmVector or rLm vaccines at an MOI of 10, as described in the legend to Supplementary Fig. 2d. In general, all rLm5Ag, rLm5AgI and rLm9Ag vaccines grew similarly to LmVector at 2 h post infection; at 4 h post infection, there were some delays in the growth of rLm5AgII and rLm9Ag clone #1 compared with LmVector; at 6 h post infection, there were delays in the growth of rLm5AgII and rLm9Ag clone #1 and clone #3; rLm5Ag grew similarly to LmVector at 2, 4, and 6 h post infection (Supplementary Fig. 2d). These results suggest that the fusion protein expression cassettes for Mtb 5AgII imposed a modest burden on its bacterial host that caused some growth delay in macrophages.

### rLm5Ag induces antigen-specific T-cell mediated immune responses in mice

**Fig. 2:**
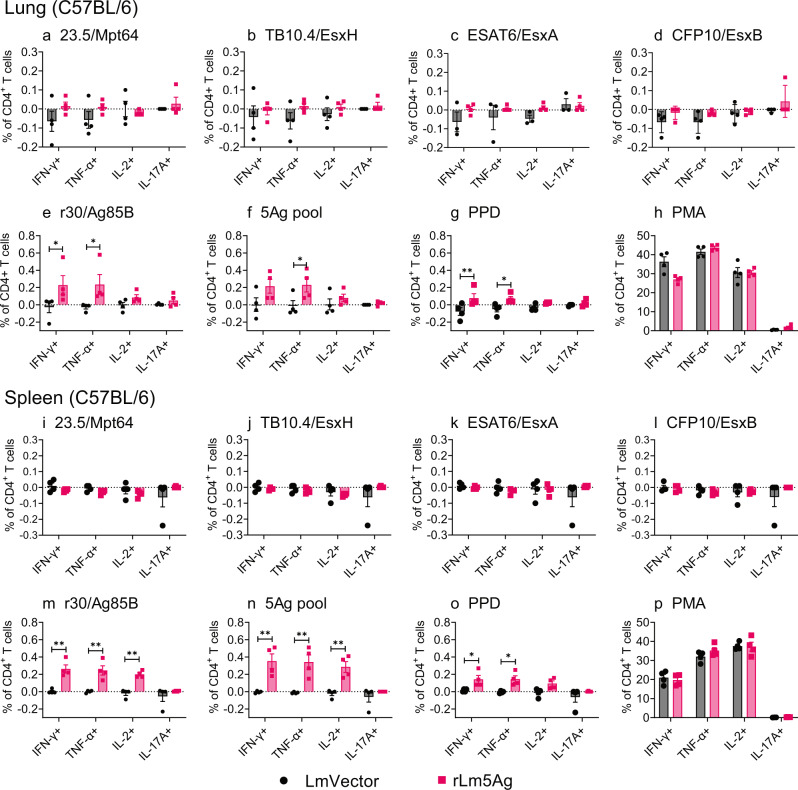
Frequency of cytokine-expressing CD4+ T cells in the lungs and spleens of C57BL/6 mice immunized three times with LmVector or rLm5Ag. C57BL/6 mice (*n* = 4/group) were immunized s.q. three times with LmVector (black) or rLm5Ag (pink) at Weeks 0, 4, and 8. Six days after the last immunization, mice were euthanized; their lungs and spleens removed; single cell suspensions prepared and stimulated with individual proteins of 23.5/Mpt64 (**a**, **i**), TB10.4/EsxH (**b**, **j**), ESAT6/EsxA (**c**, **k**), CFP10/EsxB (**d**, **l**), r30/Ag85B (**e**, **m**), or pool of 5 antigens (5Ag pool) (**f**, **n**), or PPD (**g**, **o**) in the presence of anti-CD28 monoclonal antibody for 6 h; GolgiPlug (protein transport inhibitor containing Brefeldin A) diluted in T-cell medium was added to all wells; additional cells incubated with PMA and Golgiplug for 4 h served as positive control (**h**, **p**). The cells were assayed by excluding dead cells followed by staining for surface markers of CD4 and CD8 followed by CD3 and intracellular markers of IFN-γ, TNF-α, IL-2, and IL-17A, as indicated below each panel. Frequencies of live CD4+ T cells expressing any of the four cytokines were analyzed by FlowJo 10 software. Values are the mean ± standard error of the mean (SEM). *P < 0.05; and **P < 0.01 by two-way ANOVA with Sidak’s post multiple comparisons test. A similar experiment was repeated in C57BL/6 mice.

**Fig. 3:**
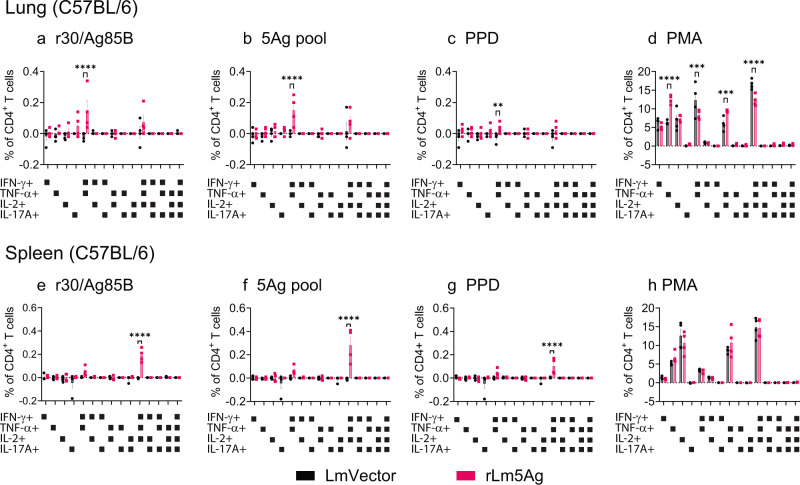
Frequency of polyfunctional cytokine-expressing CD4+ T cells in the lungs and spleens of C57BL/6 mice immunized three times with LmVector or rLm5Ag. C57BL/6 mice (*n* = 4/group, the same mice as shown in Fig. 2) were immunized three times s.q. with LmVector (black) or rLm5Ag (pink) at Weeks 0, 4, and 8. One week after the last immunization, animals were euthanized; their lung and spleen cells processed as described in the legend to Fig. 2. The lung and spleen cells were stimulated with r30/Ag85B (**a**, **e**), pool of 5 antigens (5Ag pool) (**b**, **f**), PPD (**c**, **g**), or PMA (positive control, (**d**, **h**), and assayed by intracellular cytokine staining for surface markers of CD4 and CD8 followed by CD3 and intracellular markers of IFN-γ, TNF-α, IL-2, and IL-17A. The frequencies of live CD4+ T cells producing any of the 15 possible combinations of the four cytokines (IFN-γ, TNF-α, IL-2, and IL-17A) were uniquely distinguished by using Boolean gates of FlowJo 10 software, as indicated below each panel. Values are the mean ± SEM. **P < 0.01; ***P < 0.001; and ****P < 0.0001 by two-way ANOVA with Sidak’s post multiple comparisons test.

**Fig. 4:**
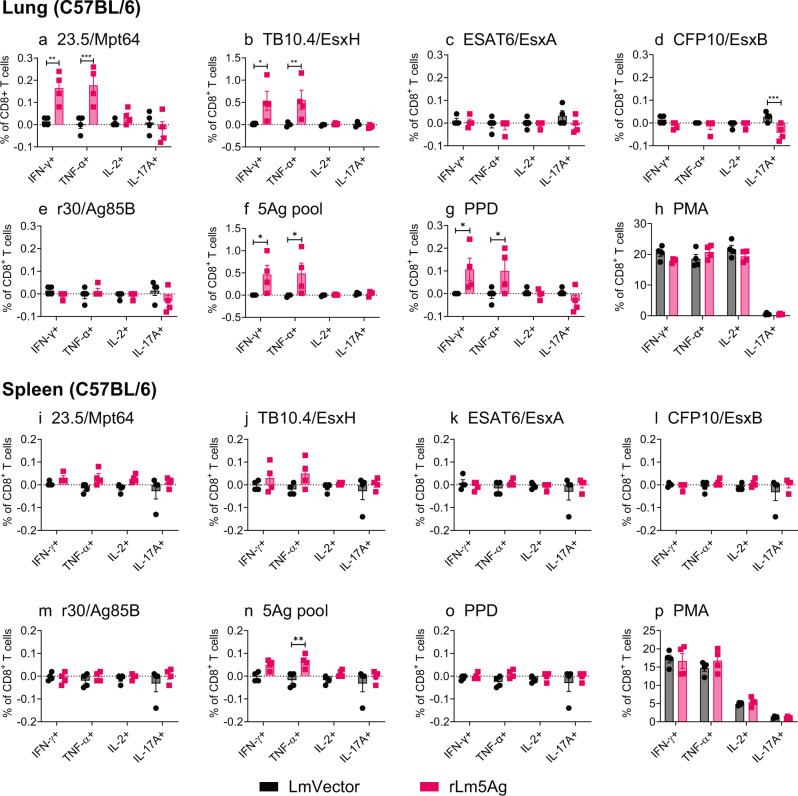
Frequency of cytokine-expressing CD8+ T cells in the lungs and spleens of C57BL/6 mice immunized three times with LmVector or rLm5Ag. C57BL/6 mice (*n* = 4/group, the same mice as shown in Fig. 2) were immunized three times s.q. with LmVector (black) or rLm5Ag (pink) at Weeks 0, 4, and 8. One week after the last immunization, animals were euthanized and their lung and spleen cells processed as described in the legend to Fig. 2. The lung and spleen cells were stimulated with individual proteins of 23.5/Mpt64 (**a**, **i**), TB10.4/EsxH (**b**, **j**), ESAT6/EsxA (**c**, **k**), CFP10/EsxB (**d**, **l**), or r30/Ag85B (**e**, **m**), or pool of 5 antigens (5Ag pool) (**f**, **n**), PPD (**g**, **o**), or PMA (**h**, **p**), and assayed by intracellular cytokine staining for surface markers of CD4 and CD8 followed by CD3 and intracellular markers of IFN-γ, TNF-α, IL-2, and IL-17A. Frequencies of CD8+ T cells expressing any of the four cytokines are analyzed by FlowJo 10 software. Values are the mean ± SEM. *P < 0.05; **P < 0.01; ***P < 0.001 by two-way ANOVA with Sidak’s post multiple comparisons test.

**Fig. 5:**
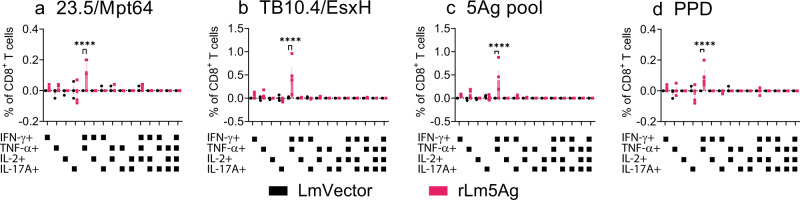
Frequency of polyfunctional cytokine-expressing CD8+ T cells in the lungs of C57BL/6 mice immunized three times with LmVector or rLm5Ag. C57BL/6 mice (*n* = 4/group, the same mice as shown in Fig. 2) were immunized three times s.q. with LmVector (black) or rLm5Ag (pink) at Weeks 0, 4, and 8. One week after the last immunization, animals were euthanized and their lung cells processed as described in the legend to Fig. 2. The lung cells were stimulated with individual proteins of 23.5/Mpt64 (**a**) or TB10.4/EsxH (**b**), or pool of 5 antigens (5Ag pool) (**c**) or PPD (**d**), and assayed by intracellular cytokine staining for surface markers of CD4 and CD8 followed by CD3 and intracellular markers of IFN-γ, TNF-α, IL-2, and IL-17A. The frequencies of live CD8+ T cells producing any of the 15 possible combinations of the four cytokines (IFN-γ, TNF-α, IL-2, and IL-17A) were uniquely distinguished by using Boolean gates of FlowJo 10 software, as indicated below each panel. Values are the mean ± SEM. ****P < 0.0001 by two-way ANOVA with Sidak’s post multiple comparisons test.

We examined the capacity of rLm5Ag to induce antigen-specific T cells and cytokine-expressing CD4+ and CD8+ T cells in the lungs and spleens of C57BL/6 mice and BALB/c mice (Figs. 2–5, Supplementary Figs. 4–7). C57BL/6 mice immunized with rLm5Ag or LmVector produced comparable frequencies of CD3+ T cells among gated lymphocytes and comparable frequencies of CD4+, CD8+ and CD4-CD8- T cells among CD3+ T cells after 22 h antigen stimulation in the lungs, and for the most part in the spleens, although differences in response to a few antigens were statistically significant (Supplementary Fig. 5).

With respect to antigen-specific cytokine-expressing T cells, the rLm5Ag-immunized C57BL/6 mice produced significantly greater frequencies of cytokine-producing CD4^+^ T cells in their lungs and spleens expressing IFN-γ, TNF-α, and/or IL-2 and polyfunctional CD4+ T cells expressing two or more cytokines among IFN-γ, TNF-α, and IL-2 in response to in vitro 6 h stimulation with Ag85B (Figs. 2e, m and 3a, e), 5Ag pool (Figs. 2f, n and 3b, f), or PPD (Figs. 2g, o and 3c, g) than mice immunized with LmVector. No significant differences in the frequencies of CD4+ T cells expressing any of the cytokines were detected after in vitro stimulation with 23.5/Mpt64 (Fig. 2a, i), TB10.4/EsxH (Fig. 2b, j), ESAT6/EsxA (Fig. 2c, k), or CFP10/EsxB (Fig. 2d, l), or as expected PMA (Fig. 2h, p). Notably, mice immunized with rLm5Ag also produced greater frequencies of CD8^+^ T cells expressing IFN-γ and TNF-α and polyfunctional CD8+ T cells expressing both cytokines in response to in vitro stimulation with 23.5/Mpt64 (Figs. 4a and 5a), TB10.4/EsxH (Figs. 4b and 5b), 5Ag pool (Figs. 4f and 5c), and PPD (Figs. 4g and 5d) than mice immunized with LmVector in the lung, and a greater frequency of CD8+ T cells expressing TNF-α in response to the 5Ag pool in the spleen (Fig. 4n). Of note, the frequencies of antigen specific cytokine-producing CD4+ and CD8+ T cells in the lungs and spleens after 22-h antigen stimulation were similar to those after 6-h stimulation. Thus, homologous priming-boosting C57BL/6 mice with rLm5Ag induces Mtb antigen-specific cytokine-expressing CD4+ and CD8+ T cells, especially Ag85B-specific CD4+ T cells expressing IFN-γ, TNF-α and IL-2, and Mpt64 and EsxH-specific CD8+ T cells expressing IFN-γ and TNF-α.

BALB/c mice immunized three times with rLm5Ag produced substantially greater (~2-5-fold) frequencies of live lung CD3+ T cells than LmVector-immunized mice after 22 h incubation whether incubated with or without antigen (*P* < 0.05–*P* < 0.001) (Supplementary Fig. 6a); of these greatly expanded numbers of CD3+ T cells, CD4+ T cell frequencies were somewhat reduced and CD8+ and CD4-CD8- T cell frequencies were somewhat increased, sometimes significantly so, whether incubated with or without antigen (*P* < 0.001–*P* < 0.0001 without antigen) (Supplementary Fig. 6b–d). In contrast, in the spleen, frequencies of CD3+, CD4+, CD8+, and CD4^−^CD8^−^ T cells were generally comparable (Supplementary Fig. 6e–h).

With respect to antigen-specific cytokine-expressing CD4+ T cells, in the spleens, BALB/c mice immunized with rLm5Ag produced significantly greater amounts of IFN-γ, TNF-α, and/or IL-17A expressing CD4+ T cells in response to 6 h in vitro stimulation with Mpt64, EsxH, EsxB, Ag85B, PPD and GI-H37RV than mice immunized with LmVector; the only antigens not inducing a significantly greater response was EsxA and, as expected, PMA (Supplementary Fig. 7, top two rows, CD4+ T cells). In contrast, with respect to antigen-specific cytokine-expressing CD8+ T cells, BALB/c mice immunized with rLm5Ag produced moderately greater frequencies of splenic CD8+ T cells expressing only IL-17A or IL-17A and IFN-γ in response to only EsxH and EsxA but not to Mpt64, EsxB, Ag85B, PPD, GI-H37RV and, as expected, PMA (Supplementary Fig. 7, bottom two rows, CD8+ T cells).

Thus, homologous priming-boosting BALB/c mice with rLm5Ag induces Mtb antigen-specific cytokine-expressing CD4+ and CD8+ T cells, where the CD4+ T cells show specificity to Mpt64, EsxH, EsxB, and Ag85B – all but EsxA – and express predominantly IFN-γ and TNF-α, and CD8+ T cells show specificity to EsxH and EsxA, but express predominantly IL-17A and IFN-γ.

Comparing C57BL/6 and BALB/c mice, immunization with rLm5Ag substantially increases the frequencies of live CD3+ T cells in the lungs of BALB/c mice (Supplementary Fig. 6a) but not C57BL/6 mice (Supplementary Fig. 5a) independent of antigen. Both mice produce antigen-specific cytokine-expressing CD4+ and CD8+ T cells, although the antigen specificity and suite of cytokines secreted differ between these two mouse strains.

### rLm5Ag and rLm9Ag induce protective immunity against aerosol challenge with virulent Mtb Erdman strain in BALB/c and C57BL/6 mice

**Fig. 6:**
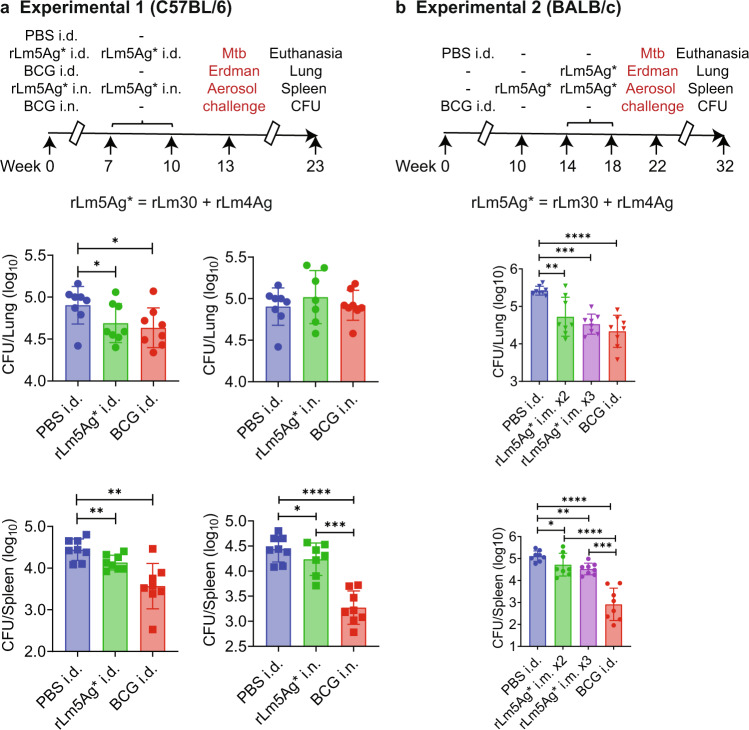
Efficacy of immunization with rLm vaccines expressing 5 Mtb antigens against Mtb aerosol challenge. **a** Top Panel: C57BL/6 mice (*n* = 8/group) were immunized i.d. with PBS (Sham), i.d. or i.n with BCG at Week 0, or immunized i.d. or i.n. twice at Weeks 7 and 10 with rLm5Ag* (rLm30 + rLm4Ag), challenged at Week 13 with aerosolized Mtb Erdman strain (average of 24 CFU delivered to the lung of each animal, as assayed at Day 1 post challenge) and euthanized at Week 23. Middle and Bottom Panels: Lungs and spleens of mice immunized i.d. (left panels) or i.n. (right panels) were assayed for organ bacterial burden. Shown are means ± SEM. Each symbol represents one mouse. *P < 0.05; **P < 0.01; ***P < 0.001 and ****P < 0.0001 by 2-tailed non-paired t-test (Prism). **b** Top Panel: BALB/c mice (8/group) were immunized i.d. with PBS or i.d. with BCG at Week 0, or immunized i.m. twice (x2) at Weeks 14 and 18 or three times (x3) at Weeks 10, 14, and 18 with rLm5Ag*. The mice were then challenged with aerosolized Mtb Erdman strain (average of 30 CFU delivered to lung of each animal, as assayed at Day 1 post challenge) at Week 22 and euthanized at Week 32. Middle and Bottom Panels: Lungs and spleens were assayed for organ bacterial burden. Shown are means ± SEM. Each symbol represents one mouse. *P < 0.05; **P < 0.01; ***P < 0.001; and ****P < 0.0001 by one-way ANOVA with Tukey’s multiple comparisons test (Prism 9.2.0).

In preliminary studies, we evaluated the protective efficacy in C57BL/6 and BALB/c mice of a combination of two rLm vaccines expressing 5 antigens - r30/Ag85B, 23.5/Mpt64, TB10.4/EsxH, ESAT6/EsxA, and CFP10/EsxB (combination of rLm30 + rLm4Ag, designated as rLm5Ag*). First, we explored i.d. and intranasal (i.n.) administration of the composite vaccine rLm5Ag* in C57BL/6 mice. In these experiments, we used BCG as a positive control against which to compare the efficacy of the Lm vaccines, as BCG consistently provides strong efficacy in animal models of TB. We immunized groups of C57BL/6 mice, 8 per group, with PBS or BCG i.d. or i.n. at Week 0, or the rLm5Ag* i.d. or i.n. three times at Weeks 0, 7, and 10, challenged all the mice with aerosolized Mtb (average of 24 CFU of the Mtb Erdman strain delivered to the lungs of each mouse, as assayed on Day 1 post-challenge) at Week 13 and euthanized the mice at Week 23 (Fig. 6a, top panel). Mice immunized i.d. three times with rLm5Ag* had significantly lower CFUs in their lungs and spleens than Sham-immunized mice (*P* < 0.05 and *P* < 0.01, respectively), not significantly different from mice immunized i.d. once with BCG (Fig. 6a, left middle and bottom panels); mice immunized i.n. once with BCG or three times with rLm5Ag* showed no reduction in bacillus burden in the lungs compared with Sham-immunized mice (Fig. 6a, right middle panel), but had significantly lower CFUs in their spleens than Sham-immunized mice (*P* < 0.0001 and *P* < 0.05, respectively) (Fig. 6a, right bottom panel).

Next, we immunized groups of 8 BALB/c mice with PBS (Sham) or BCG (positive control) i.d. at Week 0, or i.m. with the rLm5Ag* three times at Weeks 10, 14, and 18 or twice at Weeks 14 and 18, then challenged the mice at Week 22 with aerosolized Mtb (average of 30 CFU of the Mtb Erdman strain delivered to the lungs of each mouse, as assayed on Day 1 post-challenge), and euthanized the mice 10 weeks later (Week 32) to assay bacillus burden in their lungs and spleens (Fig. 6b, upper panel). As shown in Fig. 6b, middle and bottom panels, mice immunized i.m. twice or three times with rLm5Ag* had significantly lower CFUs in their lungs and spleens than Sham-immunized mice; the reduction in CFU in the lungs was comparable to that of mice immunized with BCG. Three immunizations were slightly more efficacious than two immunizations and resulted in a greater statistically significant difference from sham in both the lung and spleen.

**Fig. 7:**
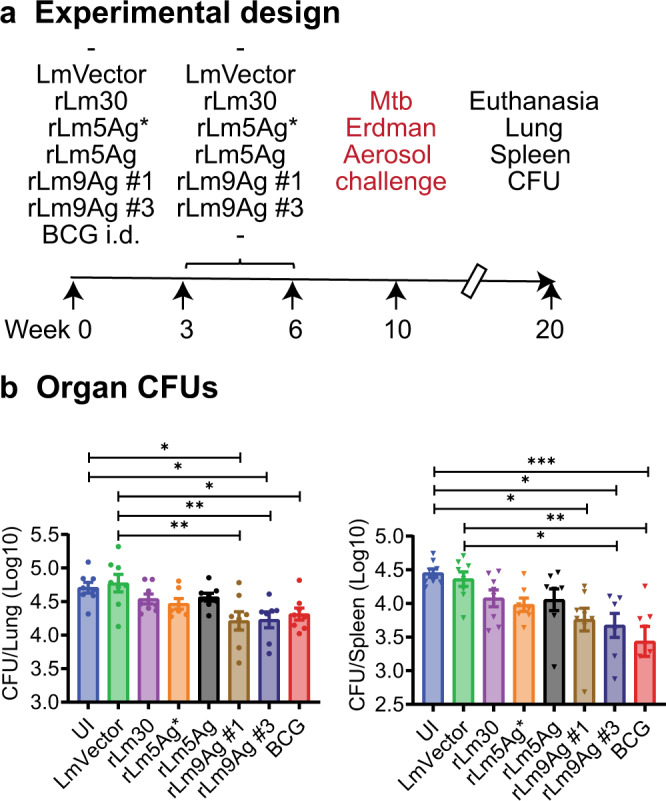
Efficacy of immunization with rLm5Ag and rLm9Ag vaccines against Mtb aerosol challenge in C57BL/6 mice. **a** C57BL/6 mice (*n* = 8/group) were unimmunized (UI), vaccinated i.d. with 5 × 10^5^ CFU BCG at Week 0, or vaccinated s.q. three times with LmVector, rLm30, rLm5Ag* (rLm30 + rLm4Ag), rLm5Ag (a single vaccine expressing the same 5 Mtb antigens as rLm5Ag*), or rLm9Ag (clones #1 and #3) at Weeks 0, 3, and 6. The mice were then challenged at Week 10 with aerosolized Mtb Erdman strain (average of 10 CFU delivered to the lungs of each animal, as assayed at Day 1 post challenge) and euthanized at Week 20. **b** Afterwards, lungs (left) and spleens (right) were removed and assayed for bacillus burdens. Shown are means ± SEM. Each symbol represents one mouse. *P < 0.05; **P < 0.01; and ***P < 0.001 by one-way ANOVA with Tukey’s multiple comparisons test (Prism 9.2.0). A similar experiment was repeated in BALB/c mice.

**Fig. 8:**
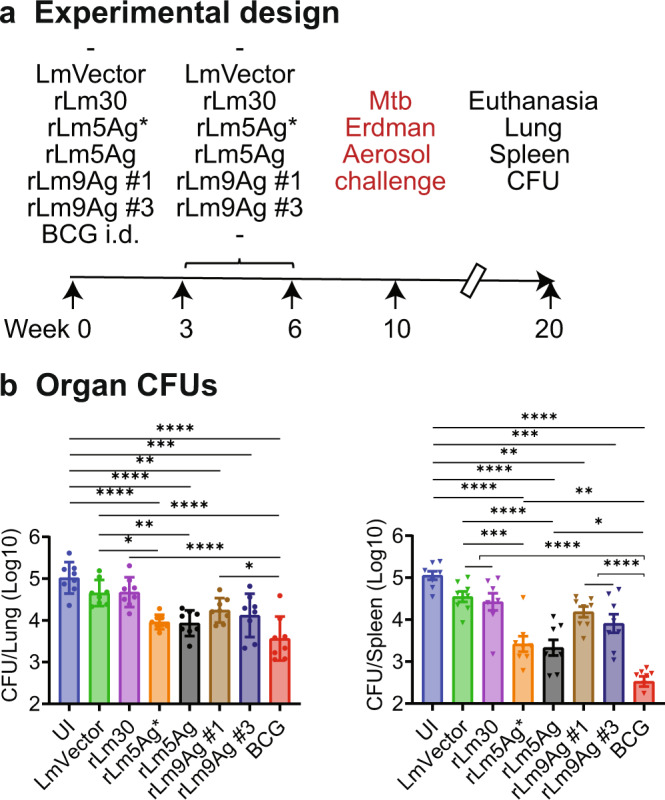
Efficacy of immunization with rLm5Ag and rLm9Ag vaccines against Mtb aerosol challenge in BALB/c mice. **a** BALB/c mice (*n* = 8/group) were unimmunized (UI), vaccinated i.d. with 5 × 10^5^ CFU BCG at Week 0, or vaccinated s.q. three times with LmVector, rLm30, rLm5Ag* (combination of rLm30 + rLm4Ag), rLm5Ag (a single vaccine expressing the same 5 Mtb antigens as rLm5Ag*), or rLm9Ag (clones #1 and #3) at Weeks 0, 3, and 6. The mice were then challenged at Week 10 with aerosolized Mtb Erdman strain (average of 10 CFU delivered to the lungs of each animal, as assayed at Day 1 post challenge) and euthanized at Week 20. **b**. Afterwards, lungs (left) and spleens (right) were removed and assayed for bacillus burdens. Shown are means ± SEM. Each symbol represents one mouse. *P < 0.05; **P < 0.01; ***P < 0.001; and ****P < 0.0001 by one-way ANOVA with Tukey’s multiple comparisons test (Prism). A similar experiment was repeated in C57BL/6 mice.

Subsequently, we performed definitive studies comparing the protective efficacy of rLm30, rLm5Ag* (combination of rLm30 + rLm4Ag), rLm5Ag (single vaccine expressing the same 5 antigens as rLm5Ag* from the *tRNA*^*arg*^ locus), and rLm9Ag (clones #1 and #3) as a standalone vaccine in both C57BL/6 and BALB/c mice immunized three times s.q., a route that was found to be both practical and efficacious against Mtb aerosol challenge in heterologous prime-boost studies involving a BCG prime and an rLm boost. We immunized groups of 8 C57BL/6 and BALB/c mice s.q. three times at Weeks 0, 3, and 6 with rLm30, rLm5Ag*, rLm5Ag, or two individual clones of rLm9Ag (rLm9Ag #1 and rLm9Ag #3); unimmunized (UI) mice or mice immunized i.d. with BCG, or three times s.q. with LmVector served as controls. Four weeks after the last immunization, mice were challenged with aerosolized Mtb (average of 10 CFU of Mtb Erdman strain delivered to the lungs of each mouse, as assayed at Day 1 post challenge). At Week 20, the mice were euthanized and organ bacillus burdens assayed (Figs. 7a and 8a).

As shown in Fig. 7, C57BL/6 mice immunized with BCG once or immunized three times with rLm30, rLm5Ag*, rLm5Ag, and both clone #1 and #3 of rLm9Ag, had lower CFUs in their lungs and spleens than sham-immunized mice and mice immunized with LmVector. Among the 4 rLm vaccines tested, rLm9Ag provided the greatest protection to C57BL/6 mice against Mtb aerosol challenge. Specifically, in the lung, mice immunized with both clones #1 and #3 of rLm9Ag had significantly lower CFU then sham-immunized mice (−0.4 and −0.5 logs, respectively (*P* < 0.05) and mice immunized with LmVector (*P* < 0.01), comparable to mice immunized with BCG (Fig. 7b, left panel). In the spleen, mice immunized with clone #1 of rLm9Ag had significantly lower CFU (−0.7 log) then sham-immunized mice (*P* < 0.05). Mice immunized with clone #3 of rLm9Ag had significantly lower CFU than both sham-immunized mice (−0.8 logs) (*P* < 0.05) and mice immunized with LmVector (*P* < 0.05), similar to and not statistically significantly different from mice immunized with BCG (Fig. 7b, right panel). These results indicate that vaccinating C57BL/6 mice with rLm vaccines comprising 5 antigens or 9 antigens as a standalone vaccine provides potent protection against aerosol challenge with virulent Mtb Erdman strain and the rLm9Ag vaccine provides protection comparable to BCG.

As shown in Fig. 8, BALB/c mice immunized with BCG once or immunized three times with rLm30, rLm5Ag*, rLm5Ag, or both clones #1 and #3 of rLm9Ag had lower CFUs in their lungs and spleens than unimmunized mice and mice immunized with LmVector. Among the 5 rLm vaccine candidates tested, rLm5Ag* (combination of rLm30 + rLm4Ag) and rLm5Ag (single vaccine) provided the best protection to BALB/c mice against Mtb aerosol challenge. Specifically, in the lung, mice immunized with rLm5Ag* and rLm5Ag had significantly lower CFU then sham-immunized mice (−1.1 and −1.1 logs, respectively) (*P* < 0.0001 and *P* < 0.0001) and mice immunized with LmVector (*P* < 0.0001 and *P* < 0.01), comparable (difference not statistically significant) to mice immunized with BCG. Mice immunized with rLm9Ag #1 and rLm9Ag #3 also had significantly lower CFU then sham-immunized mice (−0.8 and −0.9 logs, respectively) and mice immunized with LmVector (*P* < 0.01 and *P* < 0.001) in the lung (Fig. 8b, left panel). In the spleen, mice immunized with rLm5Ag* and rLm5Ag had significantly lower CFU than sham-immunized mice (−1.6 and −1.7 logs, respectively) (*P* < 0.0001 and *P* < 0.0001) and LmVector (−1.1 and −1.2 logs, respectively) (*P* < 0.001 and *P* < 0.0001). Mice immunized with rLm9Ag clone #1 and #3 also had significantly lower CFU then unimmunized mice (−0.9 and −1.1 logs, respectively) (*P* < 0.0001 and *P* < 0.0001) and mice immunized with LmVector (−0.3 and −0.6 logs, respectively) (*P* < 0.001 and *P* < 0.0001) (Fig. 8b, right panel). These results indicate that in BALB/c mice, vaccinating with rLm5Ag*, rLm5Ag, or rLm9Ag as a standalone vaccine provides potent protection against aerosol challenge with virulent Mtb Erdman strain.

### rLm9Ag induces antigen specific T-cell proliferation in guinea pigs

**Fig. 9:**
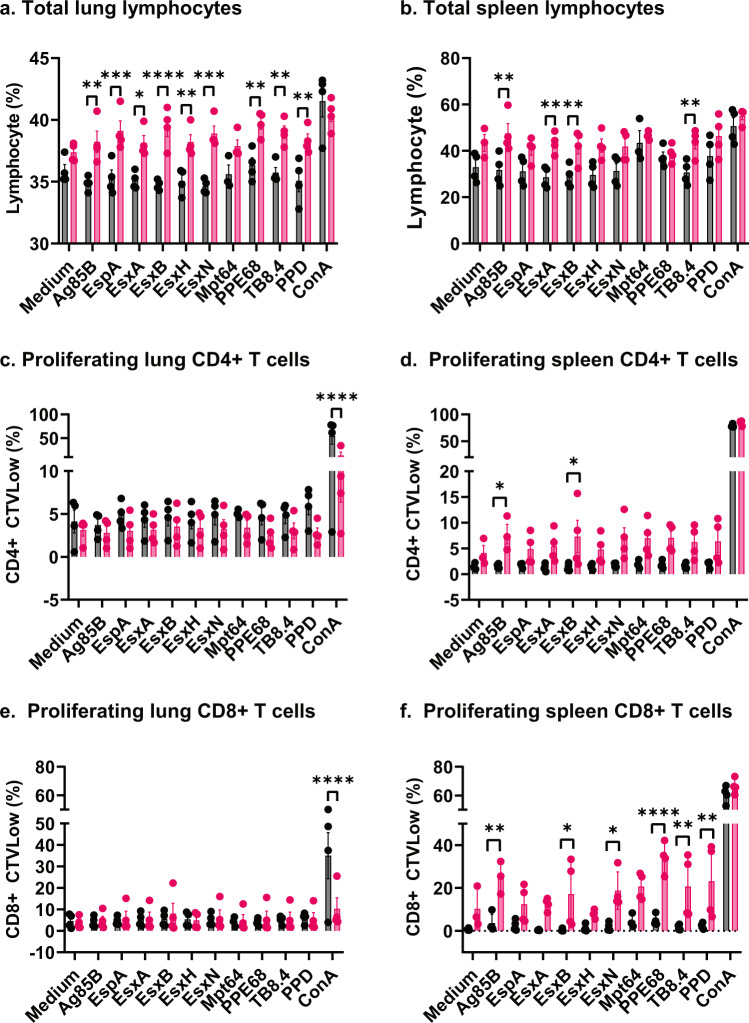
Frequencies of all lymphocytes and proliferating CD4+ and CD8+ T cells in the lungs and spleens of guinea pigs immunized with LmVector or rLm9Ag. Guinea pigs (*n* = 4/group) were immunized three times s.q. with LmVector (black) or rLm9Ag (pink) at Weeks 0, 3, and 6. Six days after the last immunization, guinea pigs were euthanized; their spleens and lungs removed; single cell suspensions of spleen and lung cells prepared and treated with Cell Tracer Violet (CTV), followed by incubation with medium alone (negative control), medium with addition of 1 µg/ml per peptide of each of the 9 Mtb antigen peptide pools plus anti-CD28 monoclonal antibody (mAb), medium with addition of 5 µg/ml of PPD protein plus anti-CD28 mAb, or medium with addition of 5 µg/ml of ConA (positive control), as indicated beneath the horizontal axis, for 4 days. Subsequently, the cells were stained with Live/dead staining dye and surface markers of CD4-PE and CD8-FITC. Total lung lymphocytes (**a**) and total spleen lymphocytes (**b**) were gated by forward and side scatter patterns; proliferating lung CD4+ T cells (**c**), proliferating spleen CD4+ T cells (**d**), proliferating lung CD8+ T cells (**e**), and proliferating spleen CD8+ T cells (**f**) were gated by corresponding antibody staining. Values are means ± SEM. *P < 0.05; **P < 0.01; ***P < 0.001; ****P < 0.0001 by two-way ANOVA with Sidak’s post multiple comparisons test (Prism 9.2.0). The experiment was repeated once with similar results.

To evaluate the capacity of the rLm9Ag vaccine to induce T-cell mediated immune responses in guinea pigs, we immunized guinea pigs with rLm9Ag or LmVector three times at Weeks 0, 3, and 6 and 6 days later evaluated responses of spleen and lung lymphocytes, CD4+ T cells, and CD8+ T cells to stimulation with Mtb peptide antigens. As shown in Fig. 9, in general, lung (Fig. 9a) and spleen (Fig. 9b) cells of guinea pigs immunized with rLm9Ag had greater frequencies of lymphocytes than guinea pigs immunized with LmVector after antigen stimulation. Specifically, in the lungs, guinea pigs immunized with rLm9Ag produced greater frequencies of lymphocytes in response to Ag85B, EspA, EsxA, EsxB, EsxH, EsxN, PE68, TB8.4 and PPD (Fig. 9a). In the spleens, guinea pigs immunized with rLm9Ag produced greater frequencies of lymphocytes in response to Ag85B, EsxA, EsxB, and TB8.4 (Fig. 9b). As expected, there were no significant differences between animals immunized with LmVector and rLm9Ag in frequencies of lymphocytes stimulated without antigen (Medium alone) or with ConA (positive control) (Fig. 9a, b). With respect to proliferating CD4+ T cells (CD4+ CTVLow) in the lung, as shown in Fig. 9c, no significant differences were detected between guinea pigs immunized with LmVector and rLm9Ag after in vitro stimulation with Mtb peptide antigens. With respect to proliferating CD4+ T cells (CD4+ CTVLow) in the spleen, as shown in Fig. 9d, guinea pigs immunized with rLm9Ag had greater frequencies of proliferating CD4+ T cells to Ag85B and EsxB than guinea pigs immunized with LmVector. With respect to proliferating CD8+ T cells (CD8+ CTVLow) in the lung, as shown in Fig. 9e, there were no significant differences between guinea pigs immunized with LmVector and rLm9Ag after in vitro stimulation with Mtb peptide antigens. With respect to proliferating CD8+ T cells (CD8 + CTVLow) in the spleen, as shown in Fig. 9f, guinea pigs immunized with rLm9Ag had greater frequencies of CD8+ T cells in response to Ag85B, EsxB, EsxN, PPE68, TB8.4, and PPD than guinea pigs immunized with LmVector.

Thus, rLm9Ag induces significantly increased lung and/or spleen cells in response to 8 of its 9 recombinant Mtb antigens, significantly increased proliferating splenic CD4+ T cells in response to Ag85B and EsxB, and significantly increased proliferating CD8+ T cells in response to 5 of its 9 Mtb antigens.

### rLm5Ag and rLm9Ag induce protective immunity against aerosol challenge with virulent Mtb Erdman strain in outbred guinea pigs

**Fig. 10:**
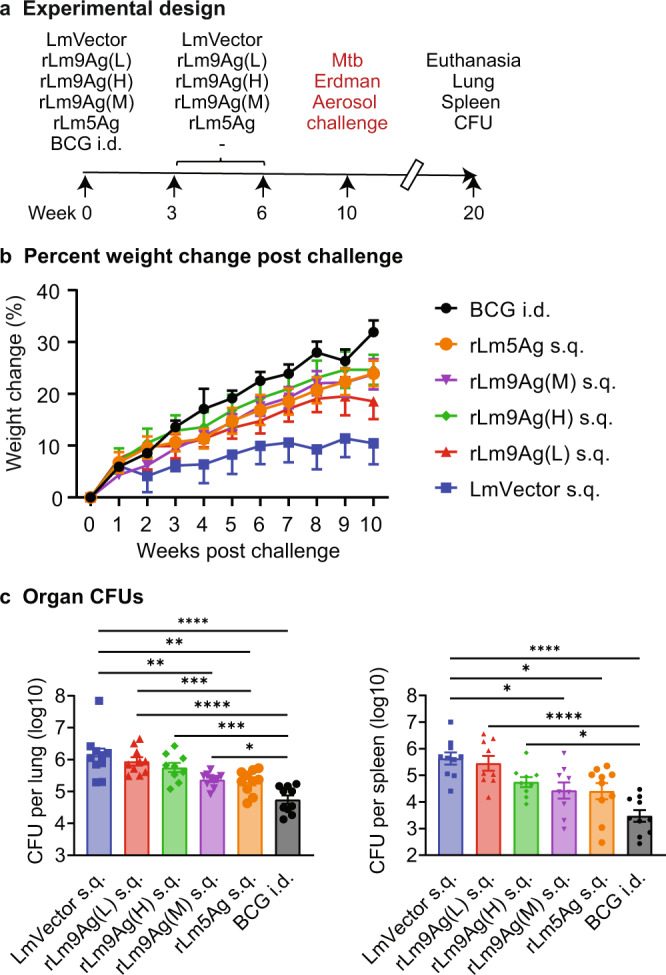
Protective efficacy of rLm9Ag and rLm5Ag vaccines against Mtb aerosol challenge in guinea pigs. **a** Experimental design. Guinea pigs (*n* = 10/group) were immunized i.d. with 10^3^ CFU BCG (Positive Control) at Week 0, or immunized s.q. three times with rLm9Ag at 10^5^ (low dose, L), 10^6^ (Medium dose, M), or 10^7^ (High Dose, H) CFU, or with 10^6^ rLm5Ag at Weeks 0, 3, and 6. The animals were then challenged at Week 10 with aerosolized Mtb Erdman strain. Afterwards, animals were monitored for signs of illness and weight change weekly for 10 weeks. At Week 10 post challenge, animals were euthanized and their spleen and right lungs were removed and assayed for bacillus burdens. **b** Percent weight change post challenge. Shown are means ± SEM. See Supplementary Fig. 8 for data points. See Supplementary Table 2 for statistical analyses comparing LmVector, rLm5Ag, rLm9Ag, and BCG groups by two-way ANOVA with Tukey’s multiple comparisons test (Prism 9.2.0). **c** Right lung (left) and spleen bacterial burden. Shown are means ± SEM. Each symbol represents one animal. *P < 0.05; **P < 0.01; ***P < 0.001; and ****P < 0.0001 by one-way ANOVA with Tukey’s multiple comparisons test.

Finally, we evaluated the capacity of the rLm5Ag and rLm9Ag vaccines to induce protective immunity against Mtb aerosol challenge in the outbred guinea pig model. As shown in Fig. 10a, we immunized guinea pigs three times s.q. at Weeks 0, 3, and 6 with the rLm multi-antigenic vaccines or LmVector, challenged them at Week 10 with aerosolized Mtb Erdman strain, subsequently monitored their weight for 10 weeks, and then euthanized them to determine CFU in their lungs and spleens; guinea pigs immunized with BCG i.d. served as a positive control. As shown in Fig. 10b, Supplementary Fig. 8 and Supplementary Table 2, guinea pigs immunized three times at Weeks 0, 3, and 6 with rLm9Ag at low, medium, and high doses, or with rLm5Ag at a medium dose gained significantly more weight 4–10 weeks post-challenge than guinea pigs immunized with LmVector (*P* < 0.05–*P* < 0.0001); the weight gain of rLm vaccinated guinea pigs was not significantly different from that of guinea pigs immunized with BCG (except guinea pigs immunized with the low dose of rLm9Ag at Week 10 post challenge). Similarly, as shown in Fig. 10c, guinea pigs immunized with low, medium, or high doses of rLm9Ag had lower Mtb bacillus burdens than guinea pigs immunized with LmVector in their lungs (0.2, 0.8, and 0.4 logs lower, respectively) (Fig. 10c, left panel) and spleens (0.2, 1.2 and 0.9 logs lower, respectively) (Fig. 10c, right panel). Similarly, guinea pigs immunized with rLm5Ag also had lower bacterial burdens than LmVector immunized guinea pigs in the lung and spleen (0.8 and 0.9 logs lower, respectively), reductions virtually equivalent to those in animals immunized with the medium dose of rLm9Ag. Guinea pigs immunized with BCG also had significantly lower Mtb bacillus burdens in their lungs (Fig. 10c left panel) (1.4 logs lower) and spleens (Fig. 10c, right panel) (2.2 logs lower) than those immunized with LmVector. Among the 3 doses of rLm9Ag tested, the medium dose (10^6^) of rLm9Ag provided the best protection against aerosol challenge with highly virulent Mtb. Thus, both the rLm5Ag and rLm9Ag vaccines induce potent protective immunity in guinea pigs.

## Discussion

Our study shows that homologous priming-boosting of inbred C57BL/6 and BALB/c mice and outbred guinea pigs with rLm5Ag (expressing a fusion protein of Mtb antigens Mpt64-EsxH-EsxA-EsxB-Ag85B) and rLm9Ag (additionally expressing Mtb antigens Mpt64- EsxN-PPE68-EspA-TB8.4) induces antigen-specific CD4+ and CD8+ T-cell mediated immunity and immunoprotection against aerosol challenge with virulent Mtb Erdman in all three animal models.

Both CD4+ and CD8+ T cells are required to control primary TB infection. CD4+ T cells help CD8+ T cells maintain effector function and prevent exhaustion, and the synergy between CD4+ and CD8+ T cells promotes the survival of mice infected with Mtb^32^. Our results show that rLm5Ag induces both CD4+ and CD8+ T cell-mediated immune responses in both C57BL/6 and BALB/c mice. In both mouse models, immunizing with rLm5Ag or rLm9Ag induces elevated frequencies of lung and/or splenic CD8+ T cells, consistent with Lm’s reputation as a potent inducer of CD8+ T cells^24^. Similarly, in guinea pigs, rLm9Ag induces proliferating antigen-specific CD4+ and CD8+ T cells. CD8+ T cells appear to play a more important role in protection against Mtb in primates than in rodents^33^. Hence, studies in rodents may underestimate the efficacy of an rLm vaccine in non-human primates and humans.

In C57BL/6 mice, rLm5Ag induced significantly elevated frequencies of antigen-specific polyfunctional CD4+ and CD8+ T cells expressing IFN-ɣ, TNF-α, and sometimes IL-2 (the CD4+ T cells in response to stimulation with Ag85B, and the CD8+ T cells in response to stimulation with Mpt64 and EsxH); the frequency of IL-17A expressing cells was not significantly elevated. In BALB/c mice, rLm5Ag induced significantly elevated frequencies of CD4+ T cells expressing IFN-ɣ and TNF-α (in response to stimulation with Mpt64, EsxH, EsxB, and Ag85B), and additionally significantly elevated frequencies of CD4+ and CD8+ T cells secreting IL17A (in response to EsxH and EsxA). Hence, these two mouse strains displayed a somewhat different CD4+ and CD8+ T cell cytokine expression profile after rLm5Ag immunization.

Among the five antigens common to both rLm5Ag and rLm9Ag, all five induced T cell responses in at least one animal model, and four induced T cell responses in multiple animal models. Antigen 85B was an especially dominant antigen, inducing T cell responses in all three animal models – cytokine-expressing CD4+ T cells in both C57BL/6 and BALB/c mice and proliferating CD4+ and CD8+ T cells in guinea pigs. EsxB also stood out, inducing cytokine-expressing CD4+ T cells in BALB/c mice and proliferating CD4+ and CD8+ T cells in guinea pigs. EsxH induced cytokine-expressing CD4+ T cells in both C57BL/6 and BALB/c mice. Finally, Mpt64 induced cytokine-expressing CD4+ T cells in BALB/c mice and CD8+ T cells in C57BL/6 mice. The rLm9 Ag vaccine, containing 4 additional Mtb antigens, was tested for immunogenicity only in guinea pigs. Three of these four new antigens – EsxN, PPE68, and TB8.4 – induced proliferating CD8+ T cells in guinea pigs.

An Lm-vectored vaccine has major advantages as a TB vaccine including i) Lm multiplies rapidly intracellularly and secrets foreign antigens into the host cell cytosol, as noted above, and then is rapidly cleared – 7 to 10 days post immunization^22^; ii) Lm-vectored vaccines with Δ*actA* Δ*inlB* deletions have an established safety profile in humans; the vaccines were well tolerated in a Phase I study^34^; iii) pre-existing immunity to Lm^35,36^ and to BCG does not deleteriously affect immunization with Lm-vectored vaccines^22^, in contrast to some virus- and mycobacterium-vectored vaccines; iv) Lm-vectored vaccines have enhanced capacity to induce both CD4+ and CD8+ T cell-mediated immune responses, and is an especially potent inducer of CD8+ T cells, as noted above; and v) Lm-vectored vaccines can be cheaply manufactured in broth medium at large scale without the need for extensive purification as with protein/adjuvant vaccines or virus-vectored vaccines grown in mammalian cells.

Although in this study we tested the Listeria vectored vaccines as standalone vaccines, we envision an rLm vaccine not as replacement vaccine for BCG but as a heterologous booster vaccine for people previously vaccinated with BCG, or in the future with an improved mycobacterial vaccine that eventually replaces BCG. Greater than 5 billion people on earth who have been vaccinated with BCG live in TB endemic areas, and hence might benefit from a heterologous TB booster vaccine. As most of these people, including adolescents and adults, would have been vaccinated with BCG in infancy, their BCG-induced immunity is likely to have largely waned by the time they were to receive such a booster vaccine many years and decades later. For this reason, as noted earlier, we considered it important to test the efficacy of our Listeria vectored vaccines as standalone vaccines. As standalone vaccines, we did not expect the rLm vaccines to be superior to BCG, which shares thousands of antigens with Mtb. However, that our studies demonstrate that the rLm vaccines are in many cases comparable in potency to BCG is noteworthy and, in our view, strongly supports the continued development of these rLm vaccines as TB booster vaccines.

In conclusion, in this study, the protective efficacy of multi-antigenic rLm TB vaccines – including rLm5Ag and rLm9Ag – was demonstrated in three rigorous animal models of pulmonary TB. This follows upon the demonstration in previous studies of the protective efficacy of a single-antigen rLm vaccine (rLm30) and multi-antigenic rLm vaccines –including rLm5Ag – as booster vaccines to enhance the level of immune protection afforded by BCG immunization. Hence, an rLm vaccine expressing multiple Mtb immunoprotective antigens has substantial promise as a new vaccine to combat the TB pandemic.

## Material and methods

### Ethics statement

All animals were maintained in a specific-pathogen-free animal facility and used according to protocols approved by the UCLA Institutional Animal Care and Use committee.

### Cell lines, bacteria, animals, protein antigens, and antibodies

Murine (J774A.1, ATCC TIB-67) monocytes were cultured as we described previously^22^. *M. bovis* BCG Tice and Mtb Erdman (ATCC 35801) strains were acquired and stocks prepared as we described previously^22^. The Listeria vector, Lm Δ*actA* ΔinlB prfA*^22,25^, derived from *Listeria monocytogenes* 10403S strain (phage-cured, DP-L4056)^37^, and recombinant Lm-vectored vaccines were grown to mid-log phase in Yeast Extract broth medium, collected by centrifugation, resuspended in PBS, titrated, and stored in 20% glycerol at −80 °C until use. Six to eight-week-old female C57BL/6 and BALB/c mice were purchased from Envigo (Indianapolis, IN) or Jackson Laboratory (Bar Harbor, Maine, USA) and three-week-old outbred male Hartley strain guinea pigs were purchased from Charles River Laboratories (Wilmington, MA, USA). The following Mtb protein reagents were obtained through BEI Resources, NIAID, NIH: Ag85B (Gene Rv1886c), Purified Native Protein from Strain H37Rv, NR-14857; ESAT-6, Recombinant Protein Reference Standard, NR-49424; CFP-10, Recombinant Protein Reference Standard, NR-49425; Mpt64, Recombinant Protein Reference Standard, NR-44102; and GI-H37RV, Mtb, Strain H37Rv, Gamma-Irradiated Whole Cells, NR14819. The Mtb protein EsxH/TB10.4 (gene Rv0288) was obtained from Aeras (formerly Rockville, Maryland, United States). Rabbit polyclonal antibody to ActAN (AK18, lot D4698) was obtained courtesy of Justin Skoble and Pete Lauer; rabbit polyclonal antibody to TB10.4 was obtained from Aeras (formerly Rockville, Maryland, United States); monoclonal antibody to Lm P60 (P6007, Lot AG-20A-0022-C100) was purchased from AdipoGen (San Diego, United States); and monoclonal antibody to β -actin (A5441) was purchased from Sigma (St. Louis, United States).

### Construction of Lm-vectored multi-antigenic vaccines

We constructed Lm-vectored multi-antigenic rLm vaccine candidates using the Lm ΔactA ΔinlB prfA* vector^25^ and two Lm site-specific phage integration vectors, pPL1 and pPL2, through conjugation process, as previously described by us and others^22,23,37^. The pPL1 conjugation vector (kindly provided by Peter Lauer) utilizes the listeriophage U153 integrase and attachment site for insertion at the *comK* locus of the rLm chromosome and carries a Gram-positive chloramphenicol acetyltransferase gene; the pPL2e-derived conjugation vector [pBHE666 containing *actA* promoter and the N-terminal 100 amino acids of ActA (ActAN), kindly provided by Justin Skoble] modified from pPL2, utilizes the listeriophage PSA integrase and attachment site for insertion in the 3' end of the *tRNA*^*arg*^ gene of the rLm chromosome and carries an erythromycin resistance gene^37^. We cloned the genes encoding Mtb proteins, optimized for expression of Mtb proteins in *Listeria monocytogenes* and purchased from DNA2.0 (currently https://www.atum.bio/) (Newark, CA), into pPL1 and pBHE666 by the restriction enzyme method and in some cases by the Electra Vector System (https://www.atum.bio/) to generate the following plasmids: pPL2e-ActAN-Mtb4Ag (for integration of ActAN-Mtb4Ag expression cassette at the *tRNA*^*arg*^ locus to construct rLm4Ag), pPL2e-ActAN-Mtb5Ag (for integration of ActAN-Mtb5Ag expression cassette at the *tRNA*^*arg*^ locus to construct rLm5Ag), pPL1-ActAN-Mtb5Ag (for integration of ActAN-Mtb5Ag expression cassette at the *comK* locus to construct rLm9Ag), and pPL2e-ActAN-Mtb5AgII (for integration of Mtb5AgII expression cassette at the *tRNA*^*arg*^ locus to construct rLm9Ag). We have deposited the sequences of these plasmids to Genbank (https://www.ncbi.nlm.nih.gov/genbank/). All molecular plasmid constructs were confirmed by restriction enzyme digestion and nucleotide sequencing. Candidate vaccines rLm30 (expressing ActAN-r30/Ag85B) (Supplementary Table 1)^22^, rLm4Ag (expressing the fusion protein ActAN-Mpt64-EsxH-EsxA-EsxB) (Supplementary Table 1), and rLm5Ag (expressing the fusion protein ActAN-Mpt64-EsxH-EsxA-EsxB-r30) (Supplementary Table 1) were constructed previously^23^ [where rLm5Ag is referred to as rLm5Ag(30)]; the Mtb protein expression cassettes in these vaccines were cloned into the pPL2e-derived vector and integrated at the *tRNA*^*arg*^ locus of the rLm chromosome. The rLm5AgII vaccine candidate (Supplementary Table 1), expressing the Mtb fusion protein of ActAN-Mpt64-EsxN-PPE68-EspA-TB8.4 from the pPL2e vector integrated at the *tRNA*^*arg*^ locus as well, was constructed similarly as described previously^22,23^. The rLm9Ag vaccine candidate (Supplementary Table 1) was constructed by integrating the pPL1-ActAN-Mtb5Ag (expressing ActAN-Mpt64-EsxH-EsxA-EsxB-r30) at the *comK locus* followed by integrating the pPL2e-ActAN-5AgII (expressing ActAN-Mpt64-EsxN-PPE68-EspA-TB8.4) at the *tRNA*^*arg*^ locus. The resultant rLm9Ag carries a total of 9 Mtb antigens with Mpt64 being a common antigen located at the N-terminus of both 5Ag and 5AgII fusion proteins. The pPL1-ActAN-Mtb5Ag conjugation vector carries a codon-optimized antigen expression cassette for the fusion protein of Mpt64(Δ1V-23A)-RP-EsxH-GGSG-EsxA-GSSGGSSG-EsxB-GSSGGSSG-Ag85B(Δ2Q-43A) (abbreviated as Mpt64-EsxH-EsxA-EsxB-r30), in which RP is a dipeptide encoded by an EagI restriction enzyme site, and GSSG and GSSGGSSG are flexible fusion protein linkers. The pPL2e-ActAN-Mtb5AgII conjugation vector carries a codon-optimized antigen expression cassette for the fusion protein of Mpt64(Δ1V-23A)-EsxN-GSSG-PPE68-GSSGGSSG-EspA(Δ111F-193L)-GSSGGSSG-TB8.4(Δ2R-28A) (abbreviated as Mpt64-EsxN-PPE68-EspA-TB8.4).

### Growth kinetics and stability of Lm-vectored multi-antigenic vaccines in vitro

The growth kinetics of rLm in broth and in murine macrophage-like cells were evaluated as described previously by us^22,23^. To assay the stability of rLm vaccines grown on agar plates, we passaged the Lm vector, rLm5Ag and rLm9Ag daily for 10 consecutive days on BHI agar plates supplemented with streptomycin, and at day 5 and day 10, we transferred 20–25 colonies of each vaccine onto BHI plates supplemented with streptomycin plus erythromycin (marker for antigen expression cassette integrated at the *tRNA*^*arg*^ locus) or streptomycin plus chloramphenicol (marker for antigen expression cassette integrated at the *comK* locus). To assay vaccine stability in macrophage-like cells, we infected monolayers of J774A.1 cells with rLm vaccines in the absence of antibiotic selection for 5.5 h; lysed the cells; serially diluted the lysates and plated them on BHI agar *s*upplemented with various antibiotics; cultured the plates at 37 °C for 2 days; and counted the colonies.

### Immunization of mice and intracellular cytokine staining of mouse spleen and lung cells

To determine the immunogenicity of rLm5Ag as a standalone vaccine, we immunized C57BL/6 and BALB/c mice, 4/group, s.q. at Weeks 0, 4, and 8 with 2 × 10^6^ Colony Forming Units (CFU) of the Lm vector (LmVector) or rLm5Ag (expressing the fusion protein of ActAN-Mpt64-EsxH-EsxA-EsxB-r30 from the *tRNA*^*arg*^ locus); euthanized the mice at 6 days post the last immunization; prepared single cell suspensions of spleen and lung cells; stimulated the single cell suspensions with various Mtb antigens for 6 h or 22 h; and assayed T-cell immunity by intracellular cytokine staining (ICS) using an eight-color flow cytometry panel to analyze simultaneously multiple cytokines at the single-cell level as described by us previously^22,23^.

### Immunization and aerosol challenge of mice with virulent Mtb Erdman strain

Groups of BALB/c or C57BL/6 mice, 8/group, were vaccinated intradermally (i.d.), intramuscularly (i.m.), intranasally (i.n.), or s.q. two or three times, 3 or 4 weeks apart, with 10^6^ CFU of LmVector or of multi-antigenic rLm vaccines; challenged 3 or 4 weeks later by exposure to an aerosol generated by a nebulizer from a 10-ml single-cell suspension of Mtb Erdman strain (2.4 × 10^4^ CFU/ml) for 30 min followed by settling for 5 min; euthanized at 10 weeks post challenge; and spleens and right lungs removed and assayed for bacillus burden as described by us previously^22^. Control mice were sham vaccinated i.d. with PBS or immunized i.d. or i.n. with 1 × 10^6^ CFU BCG at Week 0.

### Immunization of guinea pigs and lymphocyte proliferation assay of their spleen and lung cells

Guinea pigs (male Hartley), 4/group, were immunized i.d. at Weeks 0, 3, and 6 with 10^6^ CFU of the LmVector or rLm9Ag, bled and euthanized 6 days post the last immunization. Spleens and lungs were removed and single cell suspensions of spleen and lung cells prepared and stimulated with or without Mtb antigens. Lymphocyte proliferation using Flow cytometry analysis was assayed as described below.

Briefly, single cell suspensions of 1 × 10^7^ spleen and lung cells were stained with 1 µM Cell Tracer Violet (CTV, ThermoFisher, labeling cells to trace multiple generations using dye dilution by flow cytometry) for 10 min at 37 °C and washed with Phosphate buffer saline (PBS) supplemented with 5% fetal bovine serum. CTV treated cells were resuspended in T cell medium^22^, adjusted to 5 × 10^7^ cells/ml, seeded in 96-well round-bottom plates (NUNC) (5 × 10^6^ cells per 0.1 ml per well) and incubated with or without 15-mer peptide pools of each of the 9 Mtb antigens (Ag85B, EspA, EsxA, EsxB, EsxH, EsxN, Mpt64, PPE68, TB8.4) (1 µg/ml per peptide) (PepMix, JPT Peptide Technologies, Berlin, Germany) or PPD (5 µg/ml). Cells incubated with T-cell medium alone served as a negative control and cells incubated with ConA (5 µg/ml) served as a positive control. After 4-days incubation at 37 °C in a CO_2_ incubator, cells were collected, washed with PBS, and stained with LIVE/DEAD Fixable Near-IR Dead Cell (LD-NIR) (ThermoFisher), followed by staining with cell surface markers of PanT-APC (BioRad), CD4-PE (BioRad), and CD8-FITC (BioRad). Lymphocyte proliferation was analyzed as loss of CTV staining (CTVLow) using an AttuneNxt flow cytometer (ThermoFisher). Data were analyzed using FlowJo software. Initial gating of the events included lymphocytes based on forward scatter vs. side scatter pattern, followed by selection for singlet cells, live PanT+ cells, and subsequently CD4+ and CD8+ T cells. Proliferating live CD4+ and CD8+ T cells were identified by loss of CTV staining (CTVLow) on each of the cell populations. The gates for each cell population were determined by using the cells incubated without addition of antigen and verified by cells incubated with addition of ConA.

### Immunization and aerosol challenge of guinea pigs with virulent Mtb Erdman strain

Groups of guinea pigs (Hartley, male), 10/group, were vaccinated i.d. once with 10^3^ CFU BCG (positive control), or s.q. three times at Weeks 0, 3, and 6 with one dose (10^6^) of LmVector (negative control), or 3 escalating doses (10^5^, 10^6^, and 10^7^) of rLm9Ag; challenged at Week 10 by aerosol with Mtb Erdman strain (2.4 × 10^4^ CFU/ml); euthanized at Week 20; and spleens and right lungs removed and assayed for bacillus burden as described by us previously^22^.

### Vaccine stability after passaging in guinea pigs

Guinea pigs were immunized subcutaneously at the back of the neck area with 1 × 10^6^ rLm9Ag vaccine diluted in 0.1 ml PBS. At 0, 1, 2, 4, and 8 days post immunization, 2 guinea pigs were euthanized at each time point; the skin at the immunization site (~1 cm2), spleen, lung and liver of each animal were removed and homogenized in PBS; and the homogenates were serially diluted in PBS and plated onto BHI agar plates supplemented with streptomycin (200 µg/ml). The plates were incubated for 2 days at 37 °C in a CO_2_ incubator. Bacterial colonies were recovered from plates of the various tissue homogenates at 0, 1, 2, and 4 days, but not at 8 days post immunization. Recovered colonies were randomly selected and inoculated into 1 ml BHI broth plus streptomycin (200 µg/ml) and grown overnight without agitation. The bacteria were then collected from the overnight culture, lysed in SDS buffer, and subjected to SDS-PAGE and western blotting using a rabbit polyclonal antibody to Lm ActA (AK18).

### Statistics and reproducibility

Two-way ANOVA with Sidak’s multiple comparisons test was performed to determine significance in comparisons of mean frequencies of cytokine-producing CD4+ and CD8+ T cells (Figs. 2–5); one-way ANOVA with Tukey’s multiple comparisons test was used to determine mean organ CFU among mice in vaccinated and control groups (Figs. 6b, 7 and 8). Two-way ANOVA with Sidak’s multiple comparisons test was performed to determine significance in comparisons of mean frequencies of lymphocytes, CD4+, and CD8+ T cells between guinea pigs vaccinated with LmVector and those vaccinated with rLm9Ag (Fig. 9). Two-way ANOVA with Tukey’s multiple comparisons test was used to determine significance in comparisons of percent weight change between guinea pigs vaccinated with LmVector and those vaccinated with rLm9Ag (Fig. 10b) and one-way ANOVA with Tukey’s multiple comparisons test was used to determine significance in comparison of mean organ CFU among guinea pigs in vaccinated and control groups (Fig. 10c). All the statistical analyses were performed using Prism (9.2.0) software (GraphPad, San Diego, CA) except the Shapiro-Wilks tests. The Shapiro-Wilks test confirmed that all of the log scale CFU data in efficacy studies shown in Figs. 7, 8 and 10 have a normal distribution. Immunogenicity and efficacy studies were carried out in three animal models. The guinea pig immunogenicity study was repeated once.

### Reporting summary

Further information on research design is available in the Nature Portfolio Reporting Summary linked to this article.

## Supplementary information

Supplementary Information

Reporting Summary

## Data Availability

All data supporting the findings of this study are available within the article and its supplementary information files or from the corresponding author upon reasonable request.
