# Supplementary material for: Listeria-vectored multi-antigenic tuberculosis vaccine protects C57BL/6 and BALB/c mice and guinea pigs against Mycobacterium tuberculosis challenge
Source: Commun Biol. 2022 Dec 20;5:1388. doi: 10.1038/s42003-022-04345-1 (PMC9764316; doi:10.1038/s42003-022-04345-1)
Supplement: Supplementary file 1 — Supplementary Information [file 42003_2022_4345_MOESM1_ESM.pdf]

Listeria-vectored multi-antigenic tuberculosis vaccine protects C57BL/6 and BALB/c mice and guinea pigs against *Mycobacterium tuberculosis* challenge

Qingmei Jia, Saša Masleša-Galić, Susana Nava, and Marcus A. Horwitz[#]

Division of Infectious Diseases, Department of Medicine, 52-215 Center for Health Sciences, School of Medicine, University of California – Los Angeles, 10833 Le Conte Avenue, Los Angeles, CA 90095-1688, USA

Qingmei Jia: QJia@mednet.ucla.edu

Saša Masleša-Galić: sgalic@mednet.ucla.edu

Susana Nava: SusanaNava@mednet.ucla.edu

[#] Corresponding author:

Phone: (310) 206-0074; Fax: (310) 794-7156

MHorwitz@mednet.ucla.edu

Supplementary Figures

Full images for WB shown in Fig. 1b

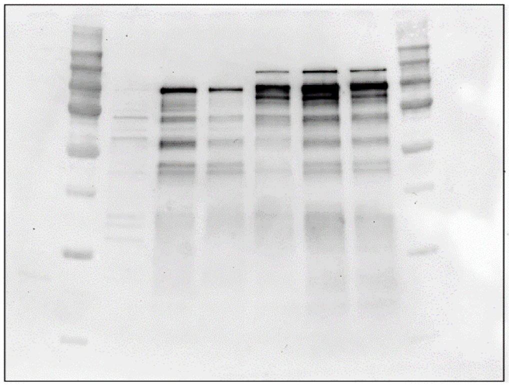

PAb to AK18

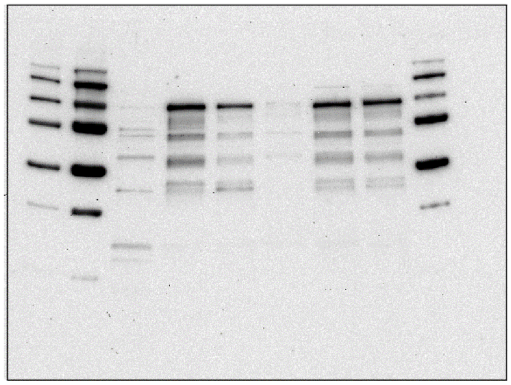

PAb to EsxH

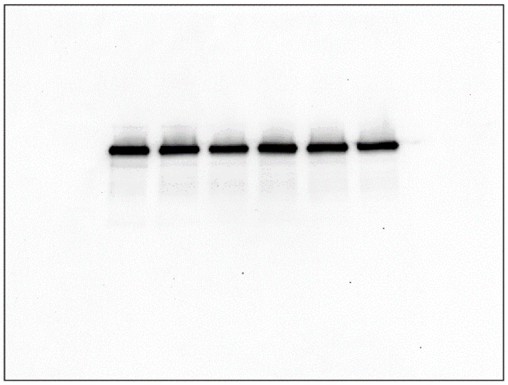

PAb to P60

Full image for WB shown in Fig. 1c

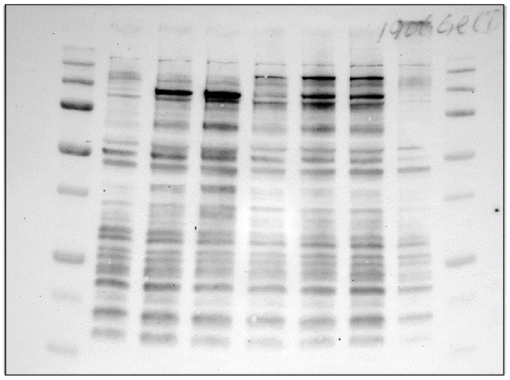

PAb to AK18

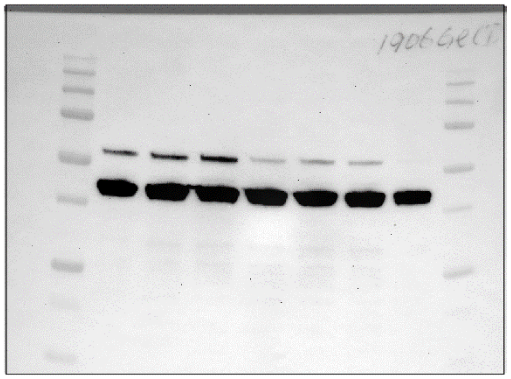

PAb to P60 + mAb to b-actin

Supplementary Figure 1. Full images of Western blotting results shown in Figure 1b and 1c.

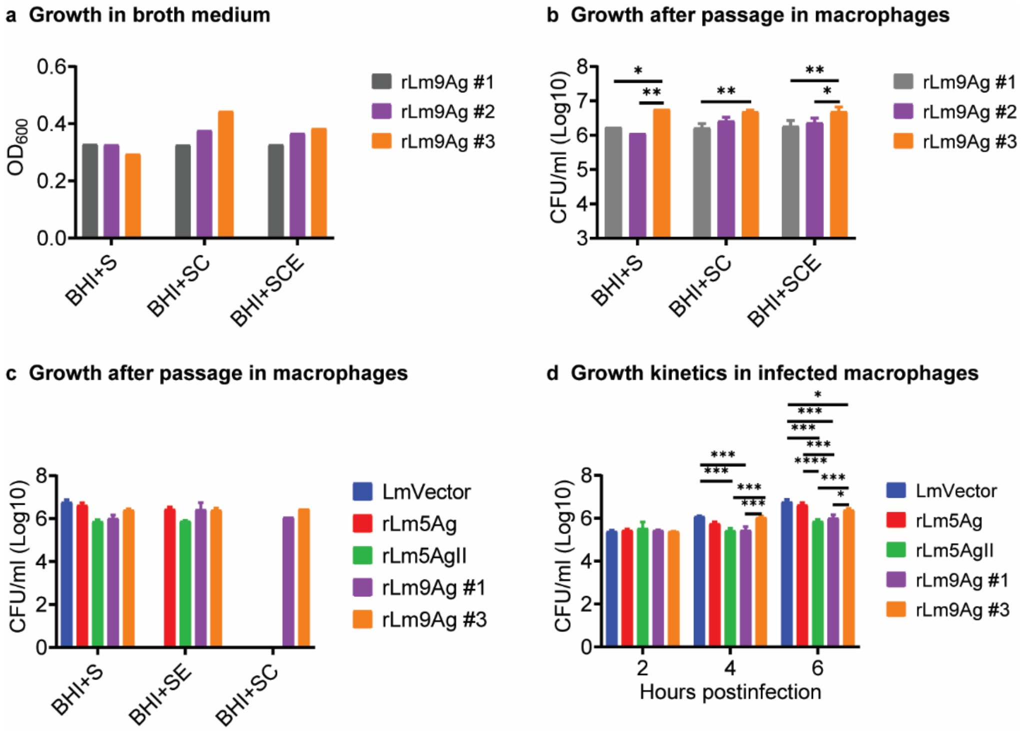

Supplementary Figure 2. The antigen expression cassettes in the rLm chromosomes are stable in the presence or absence of antibiotic selection. **a.** Each of the three clones of rLm9Ag were inoculated into Brain Heart Infusion (BHI) medium supplemented with Streptomycin (200 µg/ml) (BHI + S) (the Lm Vector is streptomycin resistant); Streptomycin (200 µg/ml) + Chloramphenicol (2.5 µg/ml) (BHI + SC); or Streptomycin (200 µg/ml) + Chloramphenicol (7.5 µg/ml) + Erythromycin (2.5 µg/ml) (BHI + SCE), as indicated below the horizontal axis. The cultures were grown overnight at 37ºC with 5% CO_2 and optical density measured at 600 nm (OD_{600}). **b & c.** Monolayers of J774A.1 cells were infected with the rLm indicated for 5.5 hours; the lysates serially diluted and plated on BHI agar supplemented with various antibiotics, as described in **a;** cultured at 37ºC for 2 days; and the colonies (CFU) enumerated. **d.** Monolayers of J774A.1 cells were infected with LmVector or the rLm indicated with an MOI of 10. At 2, 4, and 6 h post infection, cells were lysed, the lysates plated on BHI agar plates without antibiotic selection, the plates incubated for 2 days, and CFU enumerated. Values are the mean ± standard error of the mean (SEM). Values are mean log_{10} CFU ± SEM.

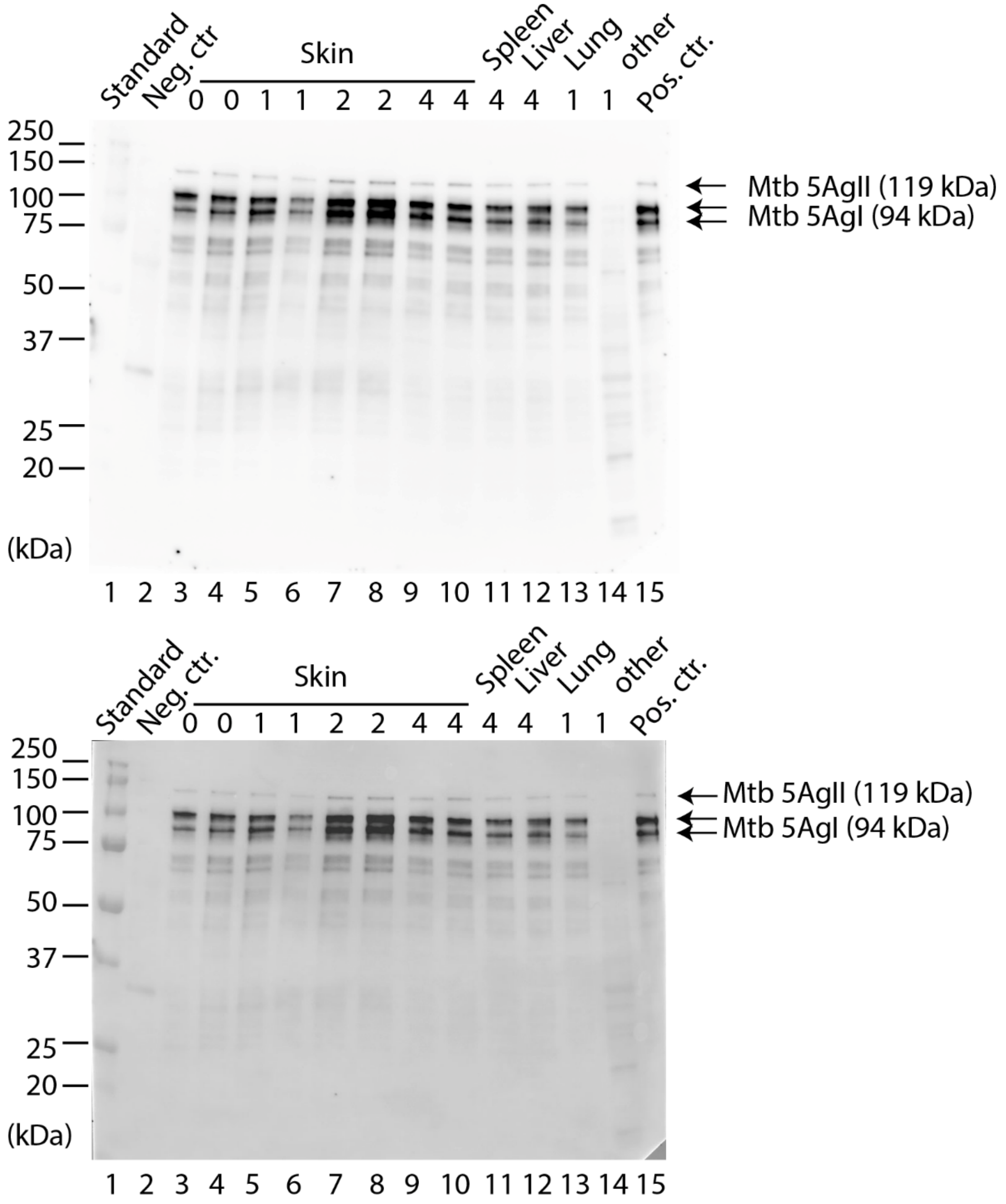

Supplementary Figure 3. Protein expression by rLm9Ag after passage in guinea pigs. Guinea pigs were immunized subcutaneously at the back of the neck area with 1 x 10^6 rLm9Ag vaccine diluted in 0.1 ml PBS. At 0, 1, 2, 4, and 8 days post immunization, 2 guinea pigs were euthanized at each time point; the skin at the immunization site (1 cm^2), spleen, lung and liver of each animal were removed and homogenized in phosphate buffered saline; and the homogenates were serially diluted and plated onto BHI agar plates supplemented with streptomycin (200 µg/ml) (BHI+Strep). The plates were incubated for 2 days at 37°C in a CO_2 incubator. Bacterial colonies were recovered from plates of the various tissue homogenates at 0, 1, 2, and 4 days, but not at 8 days post immunization. Recovered colonies were randomly selected and inoculated into 1 ml BHI broth + strep and grown overnight without agitation. The

bacteria were then collected from the overnight culture, lysed in SDS buffer, and subjected to SDS-PAGE and western blotting using a rabbit polyclonal antibody to Lm ActA (AK18). Standard: Protein standards with their masses listed to the left of the panels. Neg. ctr: Lm vector negative control. Numbers (0, 1, 2, 4) on the top of the panels: Days post immunization. Other (lane 14): a contaminant from a BHI+strep plate, also serving as a negative control. Pos. ctr: rLm1939 vaccine stock positive control. Top panel, chemiluminescence. Bottom panel, composite of chemiluminescence and stain free gel, showing the protein standards. Arrows to the right of the panels indicate expected protein bands, Mtb 5Ag (ActAN-Mpt64-EsxH-EsxA-EsxB-r30, 94 kDa doublets) and 5AgII (ActAN-Mpt64-EsxN-PPE68-EspA-TB8.4, 119 kDa). Mtb 5Ag and Mtb5AgII share a common antigen of Mpt64.

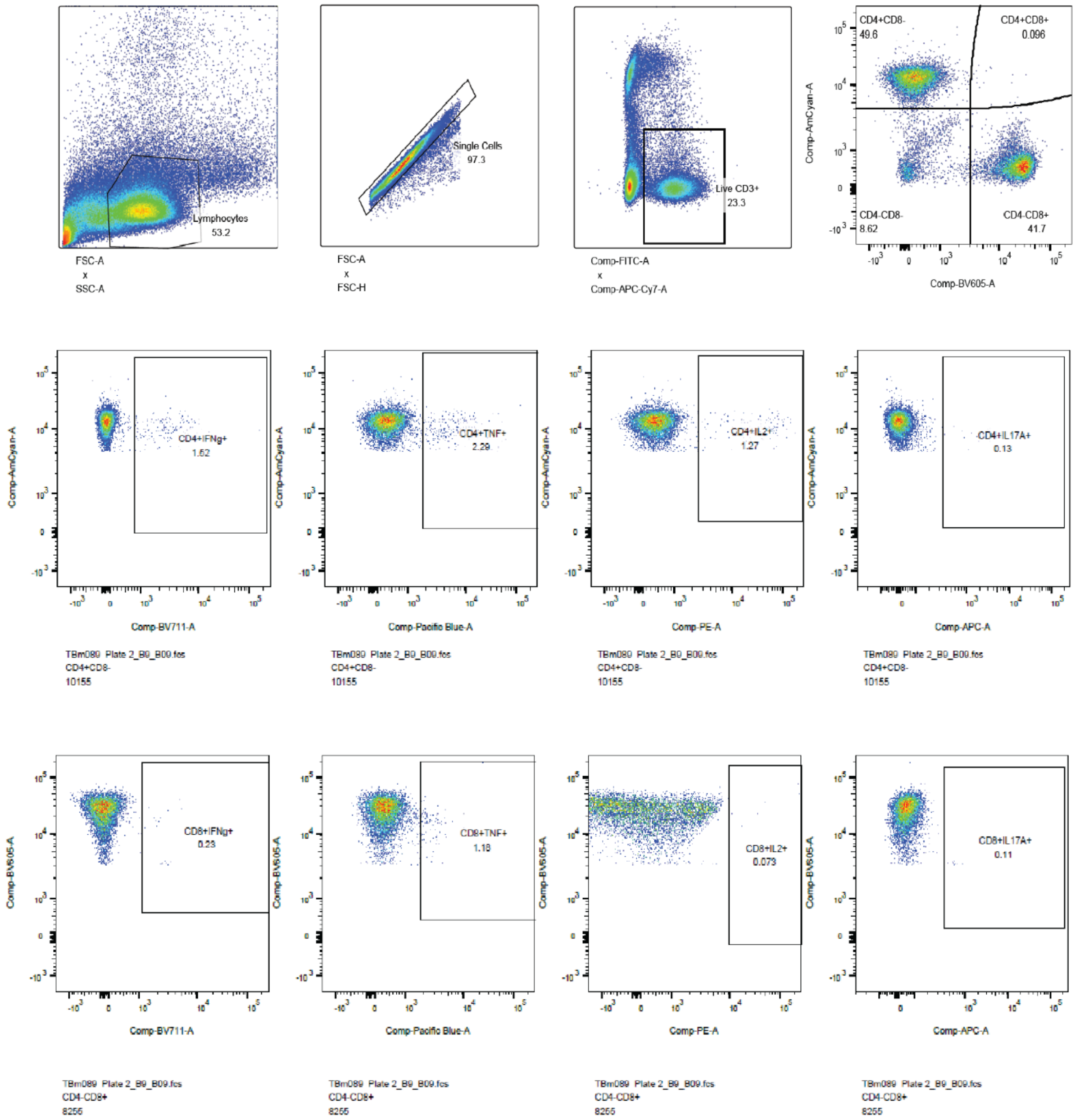

Supplementary Figure 4. Gating strategy of intracellular staining assay. C57BL/6 mice (n = 4/group) were immunized s.q. three times with LmVector or rLm5Ag at Weeks 0, 4, and 8. Six days after the last immunization, mice were euthanized; their spleens removed; single cell suspensions prepared and stimulated in T-cell medium without or with individual proteins 23.5/Mpt64, TB10.4/EsxH, ESAT6/EsxA, CFP10/EsxB, or r30/Ag85B; pool of these 5 antigens; or PPD in the presence of anti-CD28 monoclonal antibody for 2 h. Then, GolgiPlug (protein transport inhibitor containing Brefeldin A) diluted in T-cell medium was added to all wells and incubated with cells for an additional 4 h (total stimulation time of 6 h). Following *in vitro* stimulation, cells were harvested; washed with PBS; incubated with Live/Dead Fixable Near-IR Cell Stain (ThermoFisher) for 10 min at room temperature to identify dead cells; washed with Cell Staining Buffer (BioLegend) and then incubated with antibodies to CD4 (Clone RM4-5, conjugated with

Brilliant Violet 510, BD Pharmingen) and CD8a (Clone 53-6.7, conjugated with Brilliant Violet 605, BioLegend). Afterwards, cells were fixed/permeabilized with Cytofix/Cytoperm (BD BioSciences) and stained with antibodies to CD3 (clone 145-2C11, conjugated with Alexa Fluor 488, BD Pharmingen), IFN-γ (Clone XMG1.2, conjugated with Brilliant Violet 711, BE Horizon), TNF-α (Clone MP6-XT22, conjugated with Brilliant Violet 421, BD Horizon), IL-2 (Clone JES6-5H4, conjugated with PE, BD Horizon), and IL-17A (Clone TC11-18H10.1, conjugated with Alexa Fluor 647, BD Horizon). Note that due to the internalization of CD3 in responding CD4+ T cells, cells were stained for CD3 after fixing/permeabilization. A minimum of 100,000 stained cells per sample was acquired with an LSRII-HT (BD) flow cytometer and analyzed using FlowJo software (BD BioSciences). Initial gating of total events included a lymphocyte gate, followed by selection for singlet cells and live $CD3^+$ T cells and subsequently for CD4+CD8- (CD4+) and CD4-CD8+ (CD8+) T cells (top panels). The gates for frequencies of antigen-specific IFN-γ, TNF-α, IL-2, and IL-17A producing $CD4^+$ (middle panels) and $CD8^+$ T cells (bottom panels) were determined using the unstimulated cells. Plots shown here are spleen cells from a mouse immunized with rLm5Ag and stimulated with PPD for a total of 6 h. Pseudo colors were used to differentiate various cell populations. Frequencies of each cell population are shown within each plot. Note: In LSRII-HT (BD) flow cytometer, fixable Near-IR can be detected by the same detector as for APC-Cy7; AF488 by the same detector as for FITC; BV510 by the sane detector as for Amy-Cyan; BV421 by the same detector as for Pacific blue; and AF647 by the sane detector as for APC, as shown in the plots. TBm089, TB mouse experiment 89.

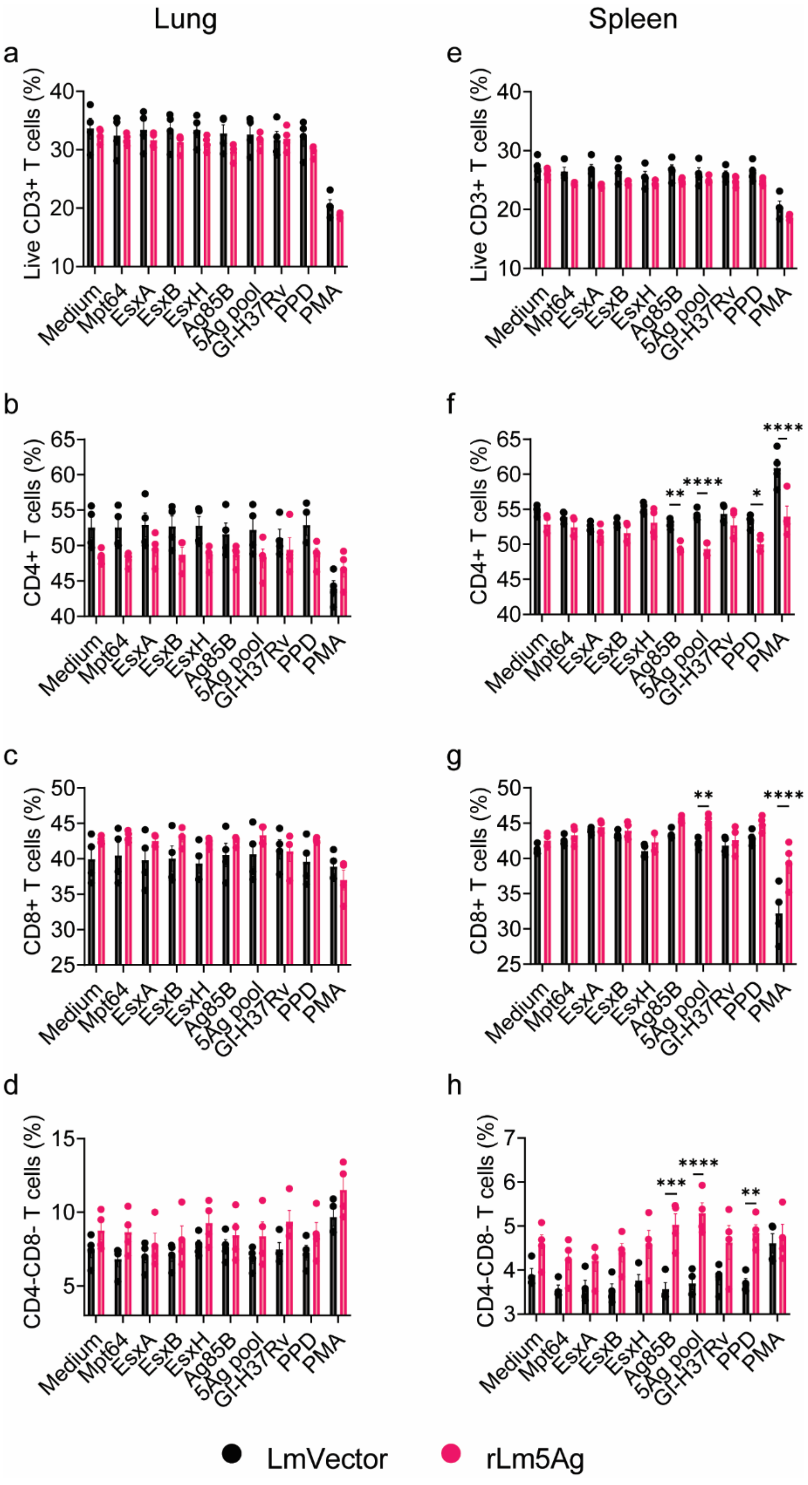

Supplementary Figure 5. Frequency of antigen-specific T cells in the lungs and spleens of C57BL/6 mice immunized three times with LmVector or rLm5Ag. C57BL/6 mice (n = 4/group, the same mice as shown in Figure 2) were immunized three times s.q. with LmVector (black) or rLm5Ag (pink) at Weeks 0, 4, and 8. At Week 9, six

days after the last immunization, animals were euthanized; their lungs and spleens removed; and single cell suspensions prepared and stimulated in T-cell medium supplemented without (Medium) or with 2μg/ml Mpt64, EsxA, EsxB, EsxH, or Ag85B, pool of 5 antigens (5Ag pool, 2μg/ml per Ag), or PPD (2μg/ml) in the presence of anti-CD28 monoclonal antibody for a total of 22 hours, as indicated below each panel. Five hours prior to harvest, GolgiPlug (protein transport inhibitor containing Brefeldin A) diluted in T-cell medium was added to all wells; additional cells incubated with PMA and Golgiplug for 5 hours served as positive control. The cells were assayed by Live/dead staining followed by staining for surface markers of CD4, CD8 and CD3 and intracellular markers of IFN-γ, TNF-α, IL-2, and IL-17A. Frequencies of live CD3+ T cells in the gated lymphocytes and frequencies of CD4+, CD8+, and CD4-CD8- T cells among live CD3+ T cells were analyzed by FlowJo 10 software. Note: frequency of CD4+CD8+ T cells among CD3+ T cells and cytokine expressing T-cells are not shown. Values are the mean ± SEM. *, $P<0.05$; **, $P<0.01$; ***, $P<0.001$; and ****, $P<0.0001$ by two-way ANOVA with Sidak's post multiple comparisons test.

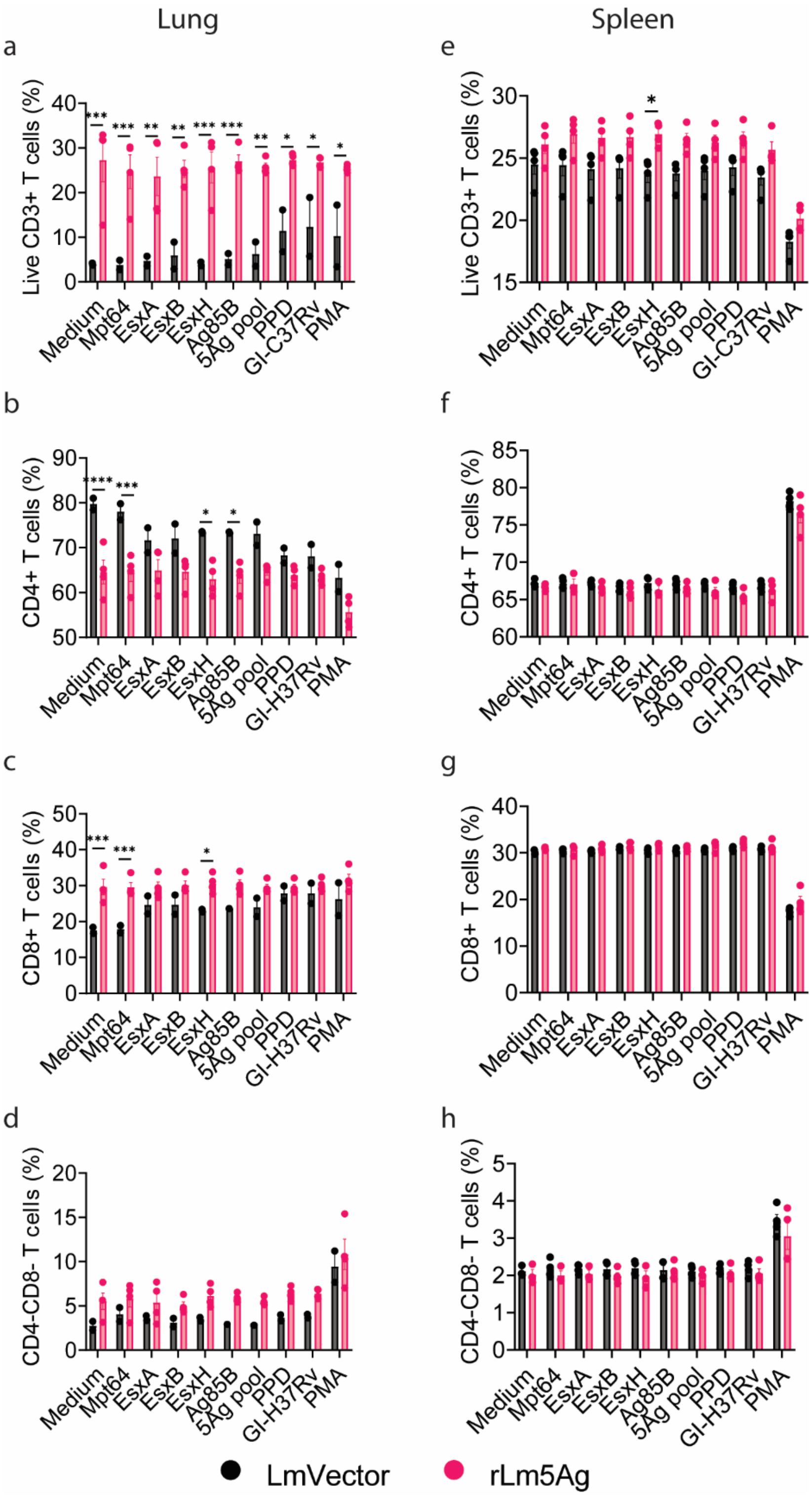

Supplementary Figure 6. Frequency of antigen-specific T cells in the lungs and spleens of BALB/c mice immunized three times with LmVector or rLm5Ag. BALB/c mice (n = 4/group) were immunized three times s.q. with LmVector (black) or rLm5Ag (pink) at Weeks 0, 4, and 8. At Week 9, six days after the last immunization, animals

were euthanized; their lungs and spleens removed, and single cell suspensions prepared and stimulated in T-cell medium supplemented without antigen (Medium) or with 2µg/ml each of Mpt64, EsxA, EsxB, EsxH, or Ag85B, pool of 5 antigens (5Ag pool, 2µg/ml per Ag), or PPD (2 µg/ml) in the presence of anti-CD28 monoclonal antibody, or with gamma-irradiated Mtb H37Rv for a total of 22 hours, as indicated below each panel. Five hours prior to harvest, GolgiPlug (protein transport inhibitor containing Brefeldin A) diluted in T-cell medium was added to all wells; additional cells incubated with PMA and Golgiplug for 5 hours served as positive control. The cells were assayed by Live/dead staining followed by staining for surface markers of CD4, CD8 and CD3 and intracellular markers of IFN-γ, TNF-α, IL-2, and IL-17A. Frequencies of live CD3+ T cells in the gated lymphocyte population, frequencies of CD4+, CD8+, and CD4-CD8- T cells among CD3+ T cells were analyzed by FlowJo 10 software. Note: frequency of CD4+CD8+ T cells among CD3+ T cells and cytokine producing T cells are not shown. Values are the mean ± SEM. Each symbol represents one animal. *, $P<0.05$; **, $P<0.01$; ***, $P<0.001$; and ****, $P<0.0001$ by two-way ANOVA with Sidak's post multiple comparisons test. The experiment was repeated once in BALB/c mice.

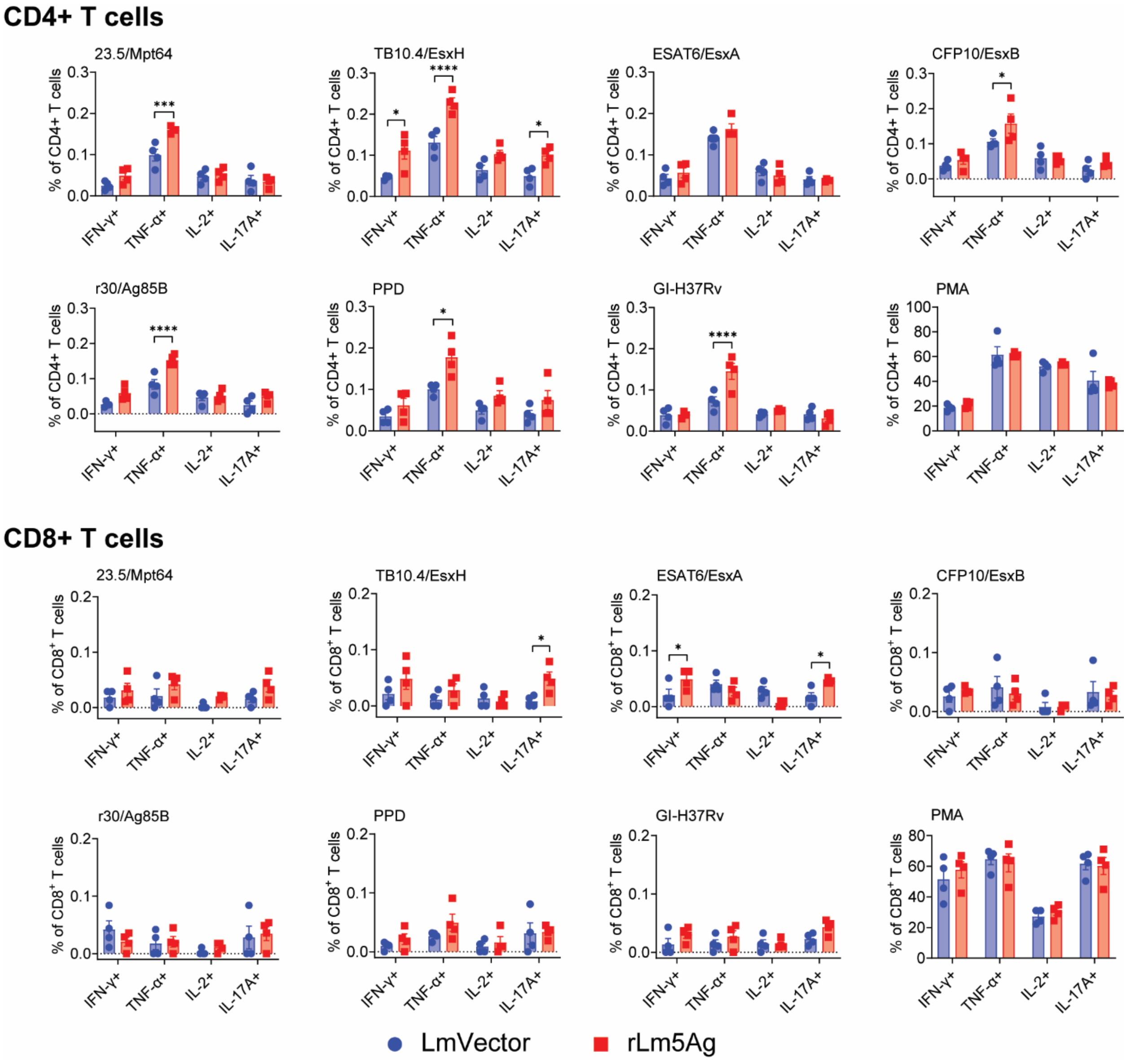

Supplementary Figure 7. Frequency of cytokine-expressing CD4+ and CD8+ T cells in the spleens of BALB/c mice immunized three times with LmVector or rLm5Ag. BALB/c mice (n = 4/group, the same mice as shown in Supplementary Figure 3) were immunized three times s.q. with LmVector (blue) or rLm5Ag (red) at Weeks 0, 4, and 8. One week after the last immunization, animals were euthanized, their lung and spleen cells stimulated with or without antigens for a total of 6 hours, and the cells subsequently processed as described in the legend to Supplementary Figure 3. The cells were assayed by intracellular cytokine staining for surface markers of CD4 and CD8 followed by CD3 and intracellular markers of IFN-γ, TNF-α, IL-2, and IL-17A. Frequencies of splenic CD4+ and CD8+ T cells expressing any of the four cytokines are analyzed by FlowJo 10 software. Values are the mean ± SEM. *, $P<0.05$; ***, $P< 0.001$; ****, $P<0.0001$ by two-way ANOVA with Sidak's post multiple comparisons test.

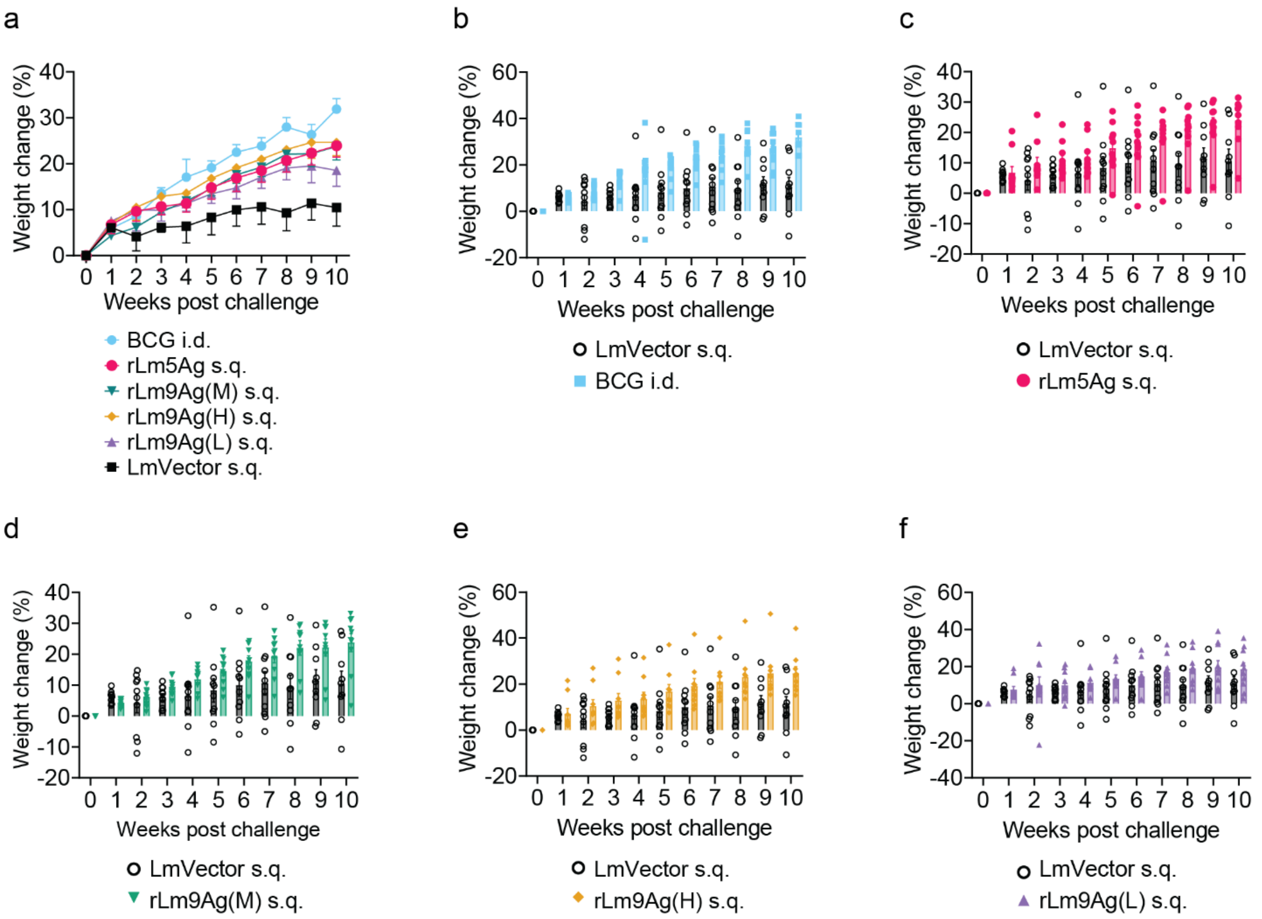

Supplementary Figure 8. Data point presentation for Figure 10b. a. The same data as shown in Fig. 10b. **b – f.** Shown are data points for panel **a** comparing vaccine group versus the LmVector group.

Supplementary Table

Supplementary Table 1. Amino acid sequences of Mtb antigens expressed by rLmMtb vaccine candidates

rLm vaccine	Antigen expression cassette	Amino acid sequence
rLm30	ActA-Ag85B	VGLNRFMRAMMVVFITANCITINPDIIFAATDSEDSSLNTDEWEEEKTEEQP SEVNTGPRYETAREVSSRDIEELEKSNKVKNTNKADLIAMLKAKAEKGGSF SRPGLPVEYLQVPSPSMGRDIKVQFQSGGNNSPAVYLLDGLRAQDDYNG WDINTPAFEWYYQSGLSIVMPVGGQSSFYSDWYSPACGKAGCQTYKWET FLTSELPQWLSANRAVKPTGSAAIGLSMAGSSAMILAAYHPQQFIYAGSLS ALLDPSQGMGPSLIGLAMGDAGGYKAADMWGPSSDPAWERNDPTQQIPK LVANNTRLWVYCGNGTPNELGGANIPAEFLENFVRSSNLKFQDAYNAAGG HNAVFNFPPNGTHSWEYWGAQLNAMKGDLQSSLGAG
rLmMtb 4Ag	ActAN-Mpt64-EsxH-GGSG-EsxA-(GSSG)2-EsxB	VGLNRFMRAMMVVFITANCITINPDIIFAATDSEDSSLNTDEWEEEKTEEQP SEVNTGPRYETAREVSSRDIEELEKSNKVKNTNKADLIAMLKAKAEKGGSA PKTYCEELKGTDTGQACQIQMSDPAYNINISLPSYYPDQKSLENYIAQTRD KFLSAATSSTPREAPYELNITSATYQSAIPPRGTQAVVLKVYQNAGGTHPTT TYKAFDWDQAYRKPITYDTLWQADTDPLPVVFPIVQGELSKQTGQQVSIAP NAGLDPVNYQNFAVTNDGVIFFFNPGELLPEAAGPTQVLVPRSAIDSMLAR PMSQIMYNYPAMLGHAGDMAGYAGTLQSLGAEIAVEQAALQSAWQGDTG ITYQAWQAQWNQAMEDLVRAYHAMSSTHEANTMAMMARDTAEAAKWG GGGSGMTEQQWNFAGIEAAASAIQGNVTSIHSLLDEGKQSLTKLAAAWGG SGSEAYQGVQQKWDATATELNNALQNLARTISEAGQAMASTEGNVTGMF AGSSGGSSGMAEMKTDAATLAQEAGNFERISGDLKTQIDQVESTAGSLQG QWRGAAGTAAQAAVVRFQEAANKQKQELDEISTNIRQAGVQYSRADEEQ QQALSSQMGF
rLmMtb 5Ag	ActAN-Mpt64-EsxH-GGSG-EsxA-(GSSG)2-EsxB-(GSSG2)-Ag85B	VGLNRFMRAMMVVFITANCITINPDIIFAATDSEDSSLNTDEWEEEKTEEQP SEVNTGPRYETAREVSSRDIEELEKSNKVKNTNKADLIAMLKAKAEKGGSM APKTYCEELKGTDTGQACQIQMSDPAYNINISLPSYYPDQKSLENYIAQTR DKFLSAATSSTPREAPYELNITSATYQSAIPPRGTQAVVLKVYQNAGGTHP TTTYKAFDWDQAYRKPITYDTLWQADTDPLPVVFPIVQGELSKQTGQQVSI APNAGLDPVNYQNFAVTNDGVIFFFNPGELLPEAAGPTQVLVPRSAIDSML ARPMSQIMYNYPAMLGHAGDMAGYAGTLQSLGAEIAVEQAALQSAWQGD TGITYQAWQAQWNQAMEDLVRAYHAMSSTHEANTMAMMARDTAEAAKW GGGGSGMTEQQWNFAGIEAAASAIQGNVTSIHSLLDEGKQSLTKLAAAWG GSGSEAYQGVQQKWDATATELNNALQNLARTISEAGQAMASTEGNVTGM FAGSSGGSSGMAEMKTDAATLAQEAGNFERISGDLKTQIDQVESTAGSLQ GQWRGAAGTAAQAAVVRFQEAANKQKQELDEISTNIRQAGVQYSRADEE QQQALSSQMGFGSSGGSSGAFSRPGLPVEYLQVPSPSMGRDIKVQFQSG GNNSPAVYLLDGLRAQDDYNGWDINTPAFEWYYQSGLSIVMPVGGQSSF YSDWYSPACGKAGCQTYKWETFLTSELPQWLSANRAVKPTGSAAIGLSM AGSSAMILAAYHPQQFIYAGSLSALLDPSQGMGPSLIGLAMGDAGGYKAA DMWGPSSDPAWERNDPTQQIPKLVANNTRLWVYCGNGTPNELGGANIPA EFLENFVRSSNLKFQDAYNAAGGHNAVFNFPPNGTHSWEYWGAQLNAMK GDLQSSLGAG

rLmMtb 5AgII	ActAN-Mpt64-EsxN-(GGSG)-PPE68-(GSSG)2-EspA-(GSSG)2-TB8.4	VGLNRFMRAMMVVFITANCITINPDIIFAATDSEDSSLNTDEWEEEKTEEQP<br>SEVNTGPRYETAREVSSRDIEELEKSNKVKNTNKADLIAMLKAKAEKGGSM<br>APKTYCEELKGTDTGQACQIQMSDPAYNINISLPSYYPDQKSLENYIAQTR<br>DKFLSAATSSTPREAPYELNITSATYQSAIPPRGTQAVVLKVYQNAGGTHP<br>TTTYKAFDWDQAYRKPITYDTLWQADTDPLPVVFPIVQGELSKQTGQQVSI<br>APNAGLDPVNYQNFAVTNDGVIFFFNPGELLPEAAGPTQVLVPRSAIDSML<br>AMTINYQFGDVDAHGAMIRAQAASLEAEHQAIVRDVLAAGDFWGGAGSVA<br>CQEFITQLGRNFQVIYEQANAHGQKVQAAGNNMAQTDSAVGSSWAGGSG<br>MLWHAMPPELNTARLMAGAGPAPMLAAAAGWQTLSAALDAQAVELTARL<br>NSLGEAWTGGGSDKALAAATPMVVWLQTASTQAKTRAMQATAQAAAYTQ<br>AMATTPSLPEIAANHITQAVLTATNFFGINTIPIALTEMDYFIRMWNQAALAM<br>EVYQAETAVNTLFEKLEPMASILDPGASQSTTNPIFGMPSPGSSTPVGQLP<br>PAATQTLGQLGEMSGPMQQLTQPLQQVTSLFSQVGGTGGGNPADEEAA<br>QMGLLGTSPLSNHPLAGGSGPSAGAGLLRAESLPGAGGSLTRTPLMSQLI<br>EKPVAPSVMPAAAAGSSATGGAAPVGAGAMGQGAQSGGSTRPGLVAPA<br>PLAQEREEDDEDDWDEEDDWGSSGGSSGAMSRAFIIDPTISAIDGLYDLL<br>GIGIPNQGGILYSSLEYFEKALEELAAAFPGDGWLGSAADKYAGKNRNHVN<br>FFQELADLDRQLISLIHDQANAVQTTRDILEGAKKGLEGEVWEFITNALNGL<br>KELWDKLTGWVTGLFSRGWSNLESFFAGVPGLTGATSGLSQVTGLFGAA<br>GLSASSGLAHADSLASSASLPALAGIGGGSGFGGLPSLAQVHAASTRQAL<br>RPRADGPVGAAAEQVGGQSQLVSAQGSQGMGGPVGMGGMHPSSGASK<br>GTTTKKYSEGAAAGTEDAERAPVEADAGGGQKVLVRNVVGSSGGSSGAM<br>DPVDAVINTTCNYGQVVAALNATDPGAAAQFNASPVAQSYLRNFLAAPPP<br>QRAAMAAQLQAVPGAAQYIGLVESVAGSCNNY
rLmMtb 9Ag	ActAN-Mpt64-EsxH-GGSG-EsxA-(GSSG)2-EsxB-(GSSG2)-Ag85B	The same as for rLm5Ag
rLmMtb 9Ag	ActAN-Mpt64-EsxN-(GGSG)-PPE68-(GSSG)2-EspA-(GSSG)2-TB8.4	The same as for rLm5AgII

Supplementary Table 2. Statistical analyses comparing weight change post challenge in guinea pig groups by 2-Way ANOVA with Tukey's multiple comparisons test (Prism)

Tukey's multiple comparisons test	95.00% CI of diff.	Below threshold?	Summary	Adjusted P Value
0 Week post challenge				
BCG i.d. vs. LmVector s.q.	-9.912 to 9.912	No	ns	>0.9999
BCG i.d. vs. rLm9Ag(L) s.q.	-9.912 to 9.912	No	ns	>0.9999
BCG i.d. vs. rLm9Ag(M) s.q.	-9.912 to 9.912	No	ns	>0.9999
BCG i.d. vs. rLm9Ag(H) s.q.	-10.18 to 10.18	No	ns	>0.9999
BCG i.d. vs. rLm5Ag s.q.	-9.912 to 9.912	No	ns	>0.9999
LmVector s.q. vs. rLm9Ag(L) s.q.	-9.912 to 9.912	No	ns	>0.9999
LmVector s.q. vs. rLm9Ag(M) s.q.	-9.912 to 9.912	No	ns	>0.9999
LmVector s.q. vs. rLm9Ag(H) s.q.	-10.18 to 10.18	No	ns	>0.9999
LmVector s.q. vs. rLm5Ag s.q.	-9.912 to 9.912	No	ns	>0.9999
rLm9Ag(L) s.q. vs. rLm9Ag(M) s.q.	-9.912 to 9.912	No	ns	>0.9999
rLm9Ag(L) s.q. vs. rLm9Ag(H) s.q.	-10.18 to 10.18	No	ns	>0.9999
rLm9Ag(L) s.q. vs. rLm5Ag s.q.	-9.912 to 9.912	No	ns	>0.9999
rLm9Ag(M) s.q. vs. rLm9Ag(H) s.q.	-10.18 to 10.18	No	ns	>0.9999
rLm9Ag(M) s.q. vs. rLm5Ag s.q.	-9.912 to 9.912	No	ns	>0.9999
rLm9Ag(H) s.q. vs. rLm5Ag s.q.	-10.18 to 10.18	No	ns	>0.9999
1 Week post challenge				
BCG i.d. vs. LmVector s.q.	-10.09 to 9.734	No	ns	>0.9999
BCG i.d. vs. rLm9Ag(L) s.q.	-11.51 to 8.309	No	ns	0.9974
BCG i.d. vs. rLm9Ag(M) s.q.	-8.348 to 11.48	No	ns	0.9976
BCG i.d. vs. rLm9Ag(H) s.q.	-11.32 to 9.047	No	ns	0.9996
BCG i.d. vs. rLm5Ag s.q.	-10.74 to 9.080	No	ns	0.9999
LmVector s.q. vs. rLm9Ag(L) s.q.	-11.34 to 8.487	No	ns	0.9985
LmVector s.q. vs. rLm9Ag(M) s.q.	-8.170 to 11.65	No	ns	0.9961
LmVector s.q. vs. rLm9Ag(H) s.q.	-11.14 to 9.225	No	ns	0.9998
LmVector s.q. vs. rLm5Ag s.q.	-10.57 to 9.258	No	ns	>0.9999
rLm9Ag(L) s.q. vs. rLm9Ag(M) s.q.	-6.745 to 13.08	No	ns	0.943
rLm9Ag(L) s.q. vs. rLm9Ag(H) s.q.	-9.717 to 10.65	No	ns	>0.9999
rLm9Ag(L) s.q. vs. rLm5Ag s.q.	-9.141 to 10.68	No	ns	>0.9999
rLm9Ag(M) s.q. vs. rLm9Ag(H) s.q.	-12.88 to 7.483	No	ns	0.9742
rLm9Ag(M) s.q. vs. rLm5Ag s.q.	-12.31 to 7.516	No	ns	0.9829
rLm9Ag(H) s.q. vs. rLm5Ag s.q.	-9.879 to 10.49	No	ns	>0.9999
2 Weeks post challenge				
BCG i.d. vs. LmVector s.q.	-5.470 to 14.35	No	ns	0.7952
BCG i.d. vs. rLm9Ag(L) s.q.	-11.29 to 8.537	No	ns	0.9987
BCG i.d. vs. rLm9Ag(M) s.q.	-7.534 to 12.29	No	ns	0.9835

BCG i.d. vs. rLm9Ag(H) s.q.	-12.14 to 8.225	No	ns	0.994
BCG i.d. vs. rLm5Ag s.q.	-10.99 to 8.830	No	ns	0.9996
LmVector s.q. vs. rLm9Ag(L) s.q.	-15.73 to 4.095	No	ns	0.5469
LmVector s.q. vs. rLm9Ag(M) s.q.	-11.98 to 7.848	No	ns	0.9913
LmVector s.q. vs. rLm9Ag(H) s.q.	-16.58 to 3.783	No	ns	0.4685
LmVector s.q. vs. rLm5Ag s.q.	-15.44 to 4.388	No	ns	0.6032
rLm9Ag(L) s.q. vs. rLm9Ag(M) s.q.	-6.159 to 13.66	No	ns	0.8883
rLm9Ag(L) s.q. vs. rLm9Ag(H) s.q.	-10.77 to 9.600	No	ns	>0.9999
rLm9Ag(L) s.q. vs. rLm5Ag s.q.	-9.619 to 10.20	No	ns	>0.9999
rLm9Ag(M) s.q. vs. rLm9Ag(H) s.q.	-14.52 to 5.847	No	ns	0.8283
rLm9Ag(M) s.q. vs. rLm5Ag s.q.	-13.37 to 6.452	No	ns	0.9185
rLm9Ag(H) s.q. vs. rLm5Ag s.q.	-9.307 to 11.06	No	ns	0.9999
3 Weeks post challenge				
BCG i.d. vs. LmVector s.q.	-2.493 to 17.33	No	ns	0.2681
BCG i.d. vs. rLm9Ag(L) s.q.	-6.204 to 13.62	No	ns	0.8933
BCG i.d. vs. rLm9Ag(M) s.q.	-5.802 to 14.02	No	ns	0.8437
BCG i.d. vs. rLm9Ag(H) s.q.	-9.508 to 10.86	No	ns	>0.9999
BCG i.d. vs. rLm5Ag s.q.	-7.003 to 12.82	No	ns	0.9601
LmVector s.q. vs. rLm9Ag(L) s.q.	-13.62 to 6.201	No	ns	0.8929
LmVector s.q. vs. rLm9Ag(M) s.q.	-13.22 to 6.603	No	ns	0.9318
LmVector s.q. vs. rLm9Ag(H) s.q.	-16.93 to 3.439	No	ns	0.4071
LmVector s.q. vs. rLm5Ag s.q.	-14.42 to 5.402	No	ns	0.7845
rLm9Ag(L) s.q. vs. rLm9Ag(M) s.q.	-9.510 to 10.31	No	ns	>0.9999
rLm9Ag(L) s.q. vs. rLm9Ag(H) s.q.	-13.22 to 7.150	No	ns	0.9575
rLm9Ag(L) s.q. vs. rLm5Ag s.q.	-10.71 to 9.113	No	ns	>0.9999
rLm9Ag(M) s.q. vs. rLm9Ag(H) s.q.	-13.62 to 6.748	No	ns	0.9289
rLm9Ag(M) s.q. vs. rLm5Ag s.q.	-11.11 to 8.711	No	ns	0.9993
rLm9Ag(H) s.q. vs. rLm5Ag s.q.	-7.949 to 12.42	No	ns	0.989
4 Weeks post challenge				
BCG i.d. vs. LmVector s.q.	0.7654 to 20.59	Yes	*	0.0263
BCG i.d. vs. rLm9Ag(L) s.q.	-4.151 to 15.67	No	ns	0.5577
BCG i.d. vs. rLm9Ag(M) s.q.	-4.729 to 15.09	No	ns	0.6675
BCG i.d. vs. rLm9Ag(H) s.q.	-6.693 to 13.67	No	ns	0.9242
BCG i.d. vs. rLm5Ag s.q.	-4.190 to 15.63	No	ns	0.5652
LmVector s.q. vs. rLm9Ag(L) s.q.	-14.83 to 4.996	No	ns	0.716
LmVector s.q. vs. rLm9Ag(M) s.q.	-15.41 to 4.418	No	ns	0.6089
LmVector s.q. vs. rLm9Ag(H) s.q.	-17.37 to 2.997	No	ns	0.3334
LmVector s.q. vs. rLm5Ag s.q.	-14.87 to 4.957	No	ns	0.7091
rLm9Ag(L) s.q. vs. rLm9Ag(M) s.q.	-10.49 to 9.334	No	ns	>0.9999
rLm9Ag(L) s.q. vs. rLm9Ag(H) s.q.	-12.45 to 7.913	No	ns	0.9881
rLm9Ag(L) s.q. vs. rLm5Ag s.q.	-9.951 to 9.873	No	ns	>0.9999
rLm9Ag(M) s.q. vs. rLm9Ag(H) s.q.	-11.88 to 8.491	No	ns	0.997

rLm9Ag(M) s.q. vs. rLm5Ag s.q.	-9.373 to 10.45	No	ns	>0.9999
rLm9Ag(H) s.q. vs. rLm5Ag s.q.	-7.952 to 12.41	No	ns	0.989
5 Weeks post challenge				
BCG i.d. vs. LmVector s.q.	0.9304 to 20.75	Yes	*	0.0227
BCG i.d. vs. rLm9Ag(L) s.q.	-4.207 to 15.62	No	ns	0.5684
BCG i.d. vs. rLm9Ag(M) s.q.	-5.325 to 14.50	No	ns	0.7721
BCG i.d. vs. rLm9Ag(H) s.q.	-7.860 to 12.51	No	ns	0.9868
BCG i.d. vs. rLm5Ag s.q.	-5.551 to 14.27	No	ns	0.8076
LmVector s.q. vs. rLm9Ag(L) s.q.	-15.05 to 4.775	No	ns	0.676
LmVector s.q. vs. rLm9Ag(M) s.q.	-16.17 to 3.657	No	ns	0.4636
LmVector s.q. vs. rLm9Ag(H) s.q.	-18.70 to 1.665	No	ns	0.1607
LmVector s.q. vs. rLm5Ag s.q.	-16.39 to 3.431	No	ns	0.422
rLm9Ag(L) s.q. vs. rLm9Ag(M) s.q.	-11.03 to 8.794	No	ns	0.9995
rLm9Ag(L) s.q. vs. rLm9Ag(H) s.q.	-13.56 to 6.802	No	ns	0.9333
rLm9Ag(L) s.q. vs. rLm5Ag s.q.	-11.26 to 8.568	No	ns	0.9989
rLm9Ag(M) s.q. vs. rLm9Ag(H) s.q.	-12.45 to 7.920	No	ns	0.9883
rLm9Ag(M) s.q. vs. rLm5Ag s.q.	-10.14 to 9.686	No	ns	>0.9999
rLm9Ag(H) s.q. vs. rLm5Ag s.q.	-8.146 to 12.22	No	ns	0.9928
6 Weeks post challenge				
BCG i.d. vs. LmVector s.q.	2.643 to 22.47	Yes	**	0.0043
BCG i.d. vs. rLm9Ag(L) s.q.	-2.163 to 17.66	No	ns	0.2231
BCG i.d. vs. rLm9Ag(M) s.q.	-4.993 to 14.83	No	ns	0.7155
BCG i.d. vs. rLm9Ag(H) s.q.	-6.808 to 13.56	No	ns	0.9338
BCG i.d. vs. rLm5Ag s.q.	-4.286 to 15.54	No	ns	0.5836
LmVector s.q. vs. rLm9Ag(L) s.q.	-14.72 to 5.106	No	ns	0.7353
LmVector s.q. vs. rLm9Ag(M) s.q.	-17.55 to 2.276	No	ns	0.2379
LmVector s.q. vs. rLm9Ag(H) s.q.	-19.36 to 1.003	No	ns	0.1044
LmVector s.q. vs. rLm5Ag s.q.	-16.84 to 2.983	No	ns	0.3442
rLm9Ag(L) s.q. vs. rLm9Ag(M) s.q.	-12.74 to 7.082	No	ns	0.9645
rLm9Ag(L) s.q. vs. rLm9Ag(H) s.q.	-14.56 to 5.809	No	ns	0.823
rLm9Ag(L) s.q. vs. rLm5Ag s.q.	-12.03 to 7.789	No	ns	0.9901
rLm9Ag(M) s.q. vs. rLm9Ag(H) s.q.	-11.73 to 8.639	No	ns	0.9981
rLm9Ag(M) s.q. vs. rLm5Ag s.q.	-9.205 to 10.62	No	ns	>0.9999
rLm9Ag(H) s.q. vs. rLm5Ag s.q.	-7.932 to 12.43	No	ns	0.9886
7 Weeks post challenge				
BCG i.d. vs. LmVector s.q.	3.371 to 23.19	Yes	**	0.002
BCG i.d. vs. rLm9Ag(L) s.q.	-3.144 to 16.68	No	ns	0.3713
BCG i.d. vs. rLm9Ag(M) s.q.	-5.288 to 14.54	No	ns	0.766
BCG i.d. vs. rLm9Ag(H) s.q.	-7.307 to 13.06	No	ns	0.9661
BCG i.d. vs. rLm5Ag s.q.	-4.561 to 15.26	No	ns	0.6361
LmVector s.q. vs. rLm9Ag(L) s.q.	-16.43 to 3.397	No	ns	0.4159

LmVector s.q. vs. rLm9Ag(M) s.q.	-18.57 to 1.253	No	ns	0.1263
LmVector s.q. vs. rLm9Ag(H) s.q.	-20.59 to -0.2233	Yes	*	0.0419
LmVector s.q. vs. rLm5Ag s.q.	-17.84 to 1.980	No	ns	0.2005
rLm9Ag(L) s.q. vs. rLm9Ag(M) s.q.	-12.06 to 7.768	No	ns	0.9897
rLm9Ag(L) s.q. vs. rLm9Ag(H) s.q.	-14.07 to 6.292	No	ns	0.8843
rLm9Ag(L) s.q. vs. rLm5Ag s.q.	-11.33 to 8.495	No	ns	0.9985
rLm9Ag(M) s.q. vs. rLm9Ag(H) s.q.	-11.93 to 8.436	No	ns	0.9965
rLm9Ag(M) s.q. vs. rLm5Ag s.q.	-9.185 to 10.64	No	ns	>0.9999
rLm9Ag(H) s.q. vs. rLm5Ag s.q.	-7.709 to 12.66	No	ns	0.9825
8 Weeks post challenge				
BCG i.d. vs. LmVector s.q.	8.769 to 28.59	Yes	****	<0.0001
BCG i.d. vs. rLm9Ag(L) s.q.	-1.004 to 18.82	No	ns	0.1064
BCG i.d. vs. rLm9Ag(M) s.q.	-4.000 to 15.82	No	ns	0.5287
BCG i.d. vs. rLm9Ag(H) s.q.	-5.308 to 15.06	No	ns	0.7457
BCG i.d. vs. rLm5Ag s.q.	-2.637 to 17.19	No	ns	0.2893
LmVector s.q. vs. rLm9Ag(L) s.q.	-19.68 to 0.1386	No	ns	0.0558
LmVector s.q. vs. rLm9Ag(M) s.q.	-22.68 to -2.857	Yes	**	0.0034
LmVector s.q. vs. rLm9Ag(H) s.q.	-23.99 to -3.622	Yes	**	0.0016
LmVector s.q. vs. rLm5Ag s.q.	-21.32 to -1.494	Yes	*	0.0135
rLm9Ag(L) s.q. vs. rLm9Ag(M) s.q.	-12.91 to 6.916	No	ns	0.9548
rLm9Ag(L) s.q. vs. rLm9Ag(H) s.q.	-14.22 to 6.151	No	ns	0.8678
rLm9Ag(L) s.q. vs. rLm5Ag s.q.	-11.54 to 8.279	No	ns	0.9971
rLm9Ag(M) s.q. vs. rLm9Ag(H) s.q.	-11.22 to 9.147	No	ns	0.9997
rLm9Ag(M) s.q. vs. rLm5Ag s.q.	-8.549 to 11.27	No	ns	0.9988
rLm9Ag(H) s.q. vs. rLm5Ag s.q.	-7.784 to 12.58	No	ns	0.9848
9 Weeks post challenge				
BCG i.d. vs. LmVector s.q.	4.797 to 25.16	Yes	***	0.0004
BCG i.d. vs. rLm9Ag(L) s.q.	-3.093 to 16.73	No	ns	0.3626
BCG i.d. vs. rLm9Ag(M) s.q.	-5.738 to 14.09	No	ns	0.8349
BCG i.d. vs. rLm9Ag(H) s.q.	-8.488 to 11.88	No	ns	0.997
BCG i.d. vs. rLm5Ag s.q.	-5.863 to 13.96	No	ns	0.8519
LmVector s.q. vs. rLm9Ag(L) s.q.	-18.34 to 2.022	No	ns	0.1991
LmVector s.q. vs. rLm9Ag(M) s.q.	-20.99 to -0.6227	Yes	*	0.0302
LmVector s.q. vs. rLm9Ag(H) s.q.	-23.73 to -2.837	Yes	**	0.0041
LmVector s.q. vs. rLm5Ag s.q.	-21.11 to -0.7477	Yes	*	0.0271
rLm9Ag(L) s.q. vs. rLm9Ag(M) s.q.	-12.56 to 7.267	No	ns	0.9735
rLm9Ag(L) s.q. vs. rLm9Ag(H) s.q.	-15.31 to 5.060	No	ns	0.7033
rLm9Ag(L) s.q. vs. rLm5Ag s.q.	-12.68 to 7.142	No	ns	0.9676
rLm9Ag(M) s.q. vs. rLm9Ag(H) s.q.	-12.66 to 7.705	No	ns	0.9824
rLm9Ag(M) s.q. vs. rLm5Ag s.q.	-10.04 to 9.787	No	ns	>0.9999
rLm9Ag(H) s.q. vs. rLm5Ag s.q.	-7.830 to 12.54	No	ns	0.986

10 Weeks post challenge				
BCG i.d. vs. LmVector s.q.	10.68 to 32.21	Yes	****	<0.0001
BCG i.d. vs. rLm9Ag(L) s.q.	2.850 to 23.88	Yes	**	0.0041
BCG i.d. vs. rLm9Ag(M) s.q.	-2.388 to 18.64	No	ns	0.2347
BCG i.d. vs. rLm9Ag(H) s.q.	-3.531 to 18.01	No	ns	0.3898
BCG i.d. vs. rLm5Ag s.q.	-2.536 to 18.49	No	ns	0.2536
LmVector s.q. vs. rLm9Ag(L) s.q.	-18.26 to 2.102	No	ns	0.2085
LmVector s.q. vs. rLm9Ag(M) s.q.	-23.50 to -3.136	Yes	**	0.0028
LmVector s.q. vs. rLm9Ag(H) s.q.	-24.65 to -3.759	Yes	**	0.0016
LmVector s.q. vs. rLm5Ag s.q.	-23.65 to -3.284	Yes	**	0.0024
rLm9Ag(L) s.q. vs. rLm9Ag(M) s.q.	-15.15 to 4.674	No	ns	0.6573
rLm9Ag(L) s.q. vs. rLm9Ag(H) s.q.	-16.31 to 4.058	No	ns	0.5191
rLm9Ag(L) s.q. vs. rLm5Ag s.q.	-15.30 to 4.526	No	ns	0.6294
rLm9Ag(M) s.q. vs. rLm9Ag(H) s.q.	-11.07 to 9.296	No	ns	0.9999
rLm9Ag(M) s.q. vs. rLm5Ag s.q.	-10.06 to 9.764	No	ns	>0.9999
rLm9Ag(H) s.q. vs. rLm5Ag s.q.	-9.444 to 10.92	No	ns	>0.9999
