# Supplementary material for: Listeria-vectored multi-antigenic tuberculosis vaccine protects C57BL/6 and BALB/c mice and guinea pigs against Mycobacterium tuberculosis challenge
Source: Commun Biol. 2022 Dec 20;5:1388. doi: 10.1038/s42003-022-04345-1 (PMC9764316; doi:10.1038/s42003-022-04345-1)
Supplement: Supplementary file 2 — Reporting Summary [file 42003_2022_4345_MOESM2_ESM.pdf]

nature portfolio

Corresponding author(s): Marcus A. Horwitz

Last updated by author(s): Nov 18, 2022

Reporting Summary

Nature Portfolio wishes to improve the reproducibility of the work that we publish. This form provides structure for consistency and transparency in reporting. For further information on Nature Portfolio policies, see our Editorial Policies and the Editorial Policy Checklist.

Statistics

For all statistical analyses, confirm that the following items are present in the figure legend, table legend, main text, or Methods section.

n/a	Confirmed	
☐	☒	The exact sample size (n) for each experimental group/condition, given as a discrete number and unit of measurement
☐	☒	A statement on whether measurements were taken from distinct samples or whether the same sample was measured repeatedly
☐	☒	The statistical test(s) used AND whether they are one- or two-sided<br>*Only common tests should be described solely by name; describe more complex techniques in the Methods section.*
☐	☒	A description of all covariates tested
☐	☒	A description of any assumptions or corrections, such as tests of normality and adjustment for multiple comparisons
☐	☒	A full description of the statistical parameters including central tendency (e.g. means) or other basic estimates (e.g. regression coefficient) AND variation (e.g. standard deviation) or associated estimates of uncertainty (e.g. confidence intervals)
☐	☐	For null hypothesis testing, the test statistic (e.g. F, t, r) with confidence intervals, effect sizes, degrees of freedom and P value noted<br>*Give P values as exact values whenever suitable.*
☐	☐	For Bayesian analysis, information on the choice of priors and Markov chain Monte Carlo settings
☐	☐	For hierarchical and complex designs, identification of the appropriate level for tests and full reporting of outcomes
☐	☐	Estimates of effect sizes (e.g. Cohen's d, Pearson's r), indicating how they were calculated

Our web collection on statistics for biologists contains articles on many of the points above.

Software and code

Policy information about availability of computer code

Data collection: *Provide a description of all commercial, open source and custom code used to collect the data in this study, specifying the version used OR state that no software was used.*

Data analysis: *Provide a description of all commercial, open source and custom code used to analyse the data in this study, specifying the version used OR state that no software was used.*

For manuscripts utilizing custom algorithms or software that are central to the research but not yet described in published literature, software must be made available to editors and reviewers. We strongly encourage code deposition in a community repository (e.g. GitHub). See the Nature Portfolio guidelines for submitting code & software for further information.

Data

Policy information about availability of data

All manuscripts must include a data availability statement. This statement should provide the following information, where applicable:
- Accession codes, unique identifiers, or web links for publicly available datasets
- A description of any restrictions on data availability
- For clinical datasets or third party data, please ensure that the statement adheres to our policy

Data availability: All data supporting the findings of this study are available within the article and its supplementary information files or from the corresponding author upon reasonable request.

Human research participants

Policy information about studies involving human research participants and Sex and Gender in Research.

Reporting on sex and gender	*Use the terms sex (biological attribute) and gender (shaped by social and cultural circumstances) carefully in order to avoid confusing both terms. Indicate if findings apply to only one sex or gender; describe whether sex and gender were considered in study design whether sex and/or gender was determined based on self-reporting or assigned and methods used. Provide in the source data disaggregated sex and gender data where this information has been collected, and consent has been obtained for sharing of individual-level data; provide overall numbers in this Reporting Summary. Please state if this information has not been collected. Report sex- and gender-based analyses where performed, justify reasons for lack of sex- and gender-based analysis.*
Population characteristics	*Describe the covariate-relevant population characteristics of the human research participants (e.g. age, genotypic information, past and current diagnosis and treatment categories). If you filled out the behavioural & social sciences study design questions and have nothing to add here, write "See above."*
Recruitment	*Describe how participants were recruited. Outline any potential self-selection bias or other biases that may be present and how these are likely to impact results.*
Ethics oversight	*Identify the organization(s) that approved the study protocol.*

Note that full information on the approval of the study protocol must also be provided in the manuscript.

Field-specific reporting

Please select the one below that is the best fit for your research. If you are not sure, read the appropriate sections before making your selection.

☒ Life sciences ☐ Behavioural & social sciences ☐ Ecological, evolutionary & environmental sciences

For a reference copy of the document with all sections, see nature.com/documents/nr-reporting-summary-flat.pdf

Life sciences study design

All studies must disclose on these points even when the disclosure is negative.

Sample size	Sample size was determined from previous experience with similar experiments that gave statistically significant differences between vaccinated and control groups.
Data exclusions	No data exclusion.
Replication	The immunology studies conducted in C57BL and BALB/c mice were repeated once in the same mouse strain. The efficacy studies on rLm9Ag vaccine conducted in C57BL and BALB/c mice and in guinea pigs were not repeated in the same animal species.
Randomization	Animals were randomly assigned to vaccine groups and control groups.
Blinding	In all the studies conducted in mice and in guinea pigs, animals were randomly allocated to different groups. Afterwards, the investigators were not blinded to group allocation due to animals being treated or not treated with different vaccines. During aerosol challenge in guinea pigs, the animals were randomly mixed and matched and pre-allocated to different challenge groups and the investigators were blinded to group allocation. The investigators were blinded to Flow Cytometry data collection for immunology studies and to the assay of organ bacterial burden for efficacy studies.

Reporting for specific materials, systems and methods

We require information from authors about some types of materials, experimental systems and methods used in many studies. Here, indicate whether each material, system or method listed is relevant to your study. If you are not sure if a list item applies to your research, read the appropriate section before selecting a response.

Materials & experimental systems

n/a	Involved in the study
☐	☒ Antibodies
☐	☒ Eukaryotic cell lines
☐	☐ Palaeontology and archaeology
☐	☒ Animals and other organisms
☐	☐ Clinical data
☐	☐ Dual use research of concern

Methods

n/a	Involved in the study
☐	☐ ChIP-seq
☐	☒ Flow cytometry
☐	☐ MRI-based neuroimaging

Antibodies

Antibodies used

Rabbit polyclonal antibody to ActAN (AK18, lot D4698) was obtained courtesy of Justin Skoble and Pete Lauer; rabbit polyclonal antibody to TB10.4 was obtained from Aeras (formerly Rockville, Maryland, United States); monoclonal antibody to Lm P60 (P6007, Lot AG-20A-0022-C100) was purchased from AdipoGen (San Diego, United States); and monoclonal antibody to β -actin (A5441) was purchased from Sigma (St. Louis, United States).

Validation

Antibodies were validated by providers/manufacturers and were able to detect proteins with the estimated molecular weight of the targeted fusion proteins.

Eukaryotic cell lines

Policy information about cell lines and Sex and Gender in Research

Cell line source(s)

Murine (J774A.1, ATCC TIB-67) monocytes

Authentication

After receiving the cells from ATTC, we propagated them for a few passages, made stocks and stored in liquid nitrogen. For each experiment, we recover one frozen vial and passage cells for up to 30 passages and check for morphological changes by light microscopy. We did not find any morphological changes of the cells.

Mycoplasma contamination

The cell line was not tested for Mycoplasma contamination.

Commonly misidentified lines
(See ICLAC register)

Name any commonly misidentified cell lines used in the study and provide a rationale for their use.

Palaeontology and Archaeology

Specimen provenance

Provide provenance information for specimens and describe permits that were obtained for the work (including the name of the issuing authority, the date of issue, and any identifying information). Permits should encompass collection and, where applicable, export.

Specimen deposition

Indicate where the specimens have been deposited to permit free access by other researchers.

Dating methods

If new dates are provided, describe how they were obtained (e.g. collection, storage, sample pretreatment and measurement), where they were obtained (i.e. lab name), the calibration program and the protocol for quality assurance OR state that no new dates are provided.

☐ Tick this box to confirm that the raw and calibrated dates are available in the paper or in Supplementary Information.

Ethics oversight

Identify the organization(s) that approved or provided guidance on the study protocol, OR state that no ethical approval or guidance was required and explain why not.

Note that full information on the approval of the study protocol must also be provided in the manuscript.

Animals and other research organisms

Policy information about studies involving animals; ARRIVE guidelines recommended for reporting animal research, and Sex and Gender in Research

Laboratory animals

Six to eight-week-old female C57BL/6 and BALB/c mice were purchased from Envigo (Indianapolis, IN) or Jackson Laboratory (Bar Harbor, Maine, USA) and three-week-old outbred male Hartley strain guinea pigs were purchased from Charles River Laboratories (Wilmington, MA, USA).

Wild animals

Not apply.

Reporting on sex

Female mice were used for easy handling during these long-term experiments. Male Hartley strain guinea pigs were used for

Reporting on sex	consistency with our previous studies.
Field-collected samples	Not apply.
Ethics oversight	All animals were maintained in a specific-pathogen-free animal facility and used according to protocols approved by the UCLA Institutional Animal Care and Use committee.

Note that full information on the approval of the study protocol must also be provided in the manuscript.

Clinical data

Policy information about clinical studies

All manuscripts should comply with the ICMJE guidelines for publication of clinical research and a completed CONSORT checklist must be included with all submissions.

Clinical trial registration	Not apply.
Study protocol	Not apply.
Data collection	Not apply.
Outcomes	Not apply.

Dual use research of concern

Policy information about dual use research of concern

Hazards

Could the accidental, deliberate or reckless misuse of agents or technologies generated in the work, or the application of information presented in the manuscript, pose a threat to:

No	Yes	
☒	☐	Public health
☒	☐	National security
☒	☐	Crops and/or livestock
☒	☐	Ecosystems
☒	☐	Any other significant area

Experiments of concern

Does the work involve any of these experiments of concern:

No	Yes	
☒	☐	Demonstrate how to render a vaccine ineffective
☒	☐	Confer resistance to therapeutically useful antibiotics or antiviral agents
☒	☐	Enhance the virulence of a pathogen or render a nonpathogen virulent
☒	☐	Increase transmissibility of a pathogen
☒	☐	Alter the host range of a pathogen
☒	☐	Enable evasion of diagnostic/detection modalities
☒	☐	Enable the weaponization of a biological agent or toxin
☒	☐	Any other potentially harmful combination of experiments and agents

ChIP-seq

Data deposition

☐ Confirm that both raw and final processed data have been deposited in a public database such as GEO.

☐ Confirm that you have deposited or provided access to graph files (e.g. BED files) for the called peaks.

Data access links<br>*May remain private before publication.*	*For "Initial submission" or "Revised version" documents, provide reviewer access links. For your "Final submission" document, provide a link to the deposited data.*
Files in database submission	*Provide a list of all files available in the database submission.*

Genome browser session
(e.g. UCSC)

Provide a link to an anonymized genome browser session for "Initial submission" and "Revised version" documents only, to enable peer review. Write "no longer applicable" for "Final submission" documents.

Methodology

Replicates

Describe the experimental replicates, specifying number, type and replicate agreement.

Sequencing depth

Describe the sequencing depth for each experiment, providing the total number of reads, uniquely mapped reads, length of reads and whether they were paired- or single-end.

Antibodies

Describe the antibodies used for the ChIP-seq experiments; as applicable, provide supplier name, catalog number, clone name, and lot number.

Peak calling parameters

Specify the command line program and parameters used for read mapping and peak calling, including the ChIP, control and index files used.

Data quality

Describe the methods used to ensure data quality in full detail, including how many peaks are at FDR 5% and above 5-fold enrichment.

Software

Describe the software used to collect and analyze the ChIP-seq data. For custom code that has been deposited into a community repository, provide accession details.

Flow Cytometry

Plots

Confirm that:

☒ The axis labels state the marker and fluorochrome used (e.g. CD4-FITC).

☒ The axis scales are clearly visible. Include numbers along axes only for bottom left plot of group (a 'group' is an analysis of identical markers).

☒ All plots are contour plots with outliers or pseudocolor plots.

☒ A numerical value for number of cells or percentage (with statistics) is provided.

Methodology

Sample preparation

Single cell suspensions were prepared and stimulated in T-cell medium without or with individual proteins 23.5/Mpt64, TB10.4/EsxH, ESAT6/EsxA, CFP10/EsxB, or r30/Ag85B; pool of these 5 antigens; or PPD in the presence of anti-CD28 monoclonal antibody for 2 h. Then, GolgiPlug (protein transport inhibitor containing Brefeldin A) diluted in T-cell medium was added to all wells and incubated with cells for an additional 4 h (total stimulation time of 6 h). Following in vitro stimulation, cells were harvested; washed with PBS; incubated with Live/Dead Fixable Near-IR Cell Stain (ThermoFisher) for 10 min at room temperature to identify dead cells; washed with Cell Staining Buffer (BioLegend) and then incubated with antibodies to CD4 (Clone RM4-5, conjugated with Brilliant Violet 510, BD Pharmingen) and CD8a (Clone 53-6.7, conjugated with Brilliant Violet 605, BioLegend). Afterwards, cells were fixed/permeabilized with Cytofix/Cytoperm (BD BioSciences) and stained with antibodies to CD3 (clone 145-2C11, conjugated with Alexa Fluor 488, BD Pharmingen), IFN-gamma (Clone XMG1.2, conjugated with Brilliant Violet 711, BE Horizon), TNF-α (Clone MP6-XT22, conjugated with Brilliant Violet 421, BD Horizon), IL-2 (Clone JES6-5H4, conjugated with PE, BD Horizon), and IL-17A (Clone TC11-18H10.1, conjugated with Alexa Fluor 647, BD Horizon). Note that due to the internalization of CD3 in responding CD4+ T cells, cells were stained for CD3 after fixing/ permeabilization. A minimum of 100,000 stained cells per sample was acquired with an LSRII-HT (BD) flow cytometer and analyzed using FlowJo software (BD BioSciences).

Instrument

A minimum of 100,000 lymphocytes per sample was acquired with an LSRII-HT (BD) flow cytometer

Software

The data were analyzed using FlowJo software.

Cell population abundance

Cells were not sorted.

Gating strategy

Initial gating of total events included a lymphocyte gate, followed by selection for singlet cells and live CD3+ T cells (Near IR-AF488+); CD4+ and CD8+ T cells were identified by CD4+ (BV510+ BV605-) and CD8+ (BV605+ BV510-) expression, respectively. The gates for frequencies of antigen-specific IFN-[illegible], IL-2, TNF-α, and IL-17A producing CD4+ and CD8+ T cells were determined by using the unstimulated cells; Boolean combinations of the four intracellular cytokine gates were used to uniquely discriminate responding cells based on their frequency with respect to cytokine production. Each cytokine-positive cell was assigned to one of the 15 possible combinations. In some cases, background frequencies of CD4+ and CD8+ T cells producing cytokines without antigen stimulation were subtracted. Note: In LSRII-HT (BD) flow cytometer, fixable Near-IR can be detected by the same detector as for APC-Cy7; AF488 by the same detector as for FITC; BV510 by the sane detector as for Amy-Cyan; BV421 by the same detector as for Pacific blue; and AF647 by the sane detector as for APC, as shown in the plots.

☒ Tick this box to confirm that a figure exemplifying the gating strategy is provided in the Supplementary Information.

Magnetic resonance imaging

Experimental design

Design type	*Indicate task or resting state; event-related or block design.*
Design specifications	*Specify the number of blocks, trials or experimental units per session and/or subject, and specify the length of each trial or block (if trials are blocked) and interval between trials.*
Behavioral performance measures	*State number and/or type of variables recorded (e.g. correct button press, response time) and what statistics were used to establish that the subjects were performing the task as expected (e.g. mean, range, and/or standard deviation across subjects).*

Acquisition

Imaging type(s)	*Specify: functional, structural, diffusion, perfusion.*
Field strength	*Specify in Tesla*
Sequence & imaging parameters	*Specify the pulse sequence type (gradient echo, spin echo, etc.), imaging type (EPI, spiral, etc.), field of view, matrix size, slice thickness, orientation and TE/TR/flip angle.*
Area of acquisition	*State whether a whole brain scan was used OR define the area of acquisition, describing how the region was determined.*

Diffusion MRI ☐ Used ☐ Not used

Preprocessing

Preprocessing software	*Provide detail on software version and revision number and on specific parameters (model/functions, brain extraction, segmentation, smoothing kernel size, etc.).*
Normalization	*If data were normalized/standardized, describe the approach(es): specify linear or non-linear and define image types used for transformation OR indicate that data were not normalized and explain rationale for lack of normalization.*
Normalization template	*Describe the template used for normalization/transformation, specifying subject space or group standardized space (e.g. original Talairach, MNI305, ICBM152) OR indicate that the data were not normalized.*
Noise and artifact removal	*Describe your procedure(s) for artifact and structured noise removal, specifying motion parameters, tissue signals and physiological signals (heart rate, respiration).*
Volume censoring	*Define your software and/or method and criteria for volume censoring, and state the extent of such censoring.*

Statistical modeling & inference

Model type and settings	*Specify type (mass univariate, multivariate, RSA, predictive, etc.) and describe essential details of the model at the first and second levels (e.g. fixed, random or mixed effects; drift or auto-correlation).*
Effect(s) tested	*Define precise effect in terms of the task or stimulus conditions instead of psychological concepts and indicate whether ANOVA or factorial designs were used.*

Specify type of analysis: ☐ Whole brain ☐ ROI-based ☐ Both

Statistic type for inference (See Eklund et al. 2016)	*Specify voxel-wise or cluster-wise and report all relevant parameters for cluster-wise methods.*
Correction	*Describe the type of correction and how it is obtained for multiple comparisons (e.g. FWE, FDR, permutation or Monte Carlo).*

Models & analysis

n/a	Involved in the study
☒	☐ Functional and/or effective connectivity
☒	☐ Graph analysis
☒	☐ Multivariate modeling or predictive analysis
